# Risk factor prediction and immune correlation analysis of cuproptosis‐related gene in osteoarthritis

**DOI:** 10.1111/jcmm.18574

**Published:** 2024-08-01

**Authors:** Jingmin Che, Xiaoli Yang, Xiangrong Zhao, Yan Li, Zhankui Jin, Cuixiang Xu

**Affiliations:** ^1^ Shaanxi Provincial Key Laboratory of Infection and Immune Diseases Shaanxi Provincial People's Hospital Xi'an Shaanxi China; ^2^ Shaanxi Engineering Research Center of Cell Immunology Shaanxi Provincial People's Hospital Xi'an Shaanxi China; ^3^ Department of Orthopedics Shaanxi Provincial People's Hospital Xi'an Shaanxi China

**Keywords:** bioinformatic analysis, chondrocyte, cuproptosis, immunoinfiltration, osteoarthritis

## Abstract

Osteoarthritis (OA) is a widespread inflammatory joint disease with significant global disability burden. Cuproptosis, a newly identified mode of cell death, has emerged as a crucial factor in various pathological conditions, including OA. In this context, our study aims to investigate the intrinsic relationship between cuproptosis‐related genes (CRGs) and OA, and assess their potential as biomarkers for OA diagnosis and treatment. Datasets from the GEO databases were analysed the differential expression of CRGs, leading to the identification of 10 key CRGs (*CDKN2A*, *DLD*, *FDX1*, *GLS*, *LIAS*, *LIPT1*, *MTF1*, *PDHA1*, *DLAT* and *PDHB*). A logistic regression analysis and calibration curves were used to show excellent diagnostic accuracy. Consensus clustering revealed two CRG patterns, with Cluster 1 indicating a closer association with OA progression. RT‐PCR confirmed a significant increase in the expression levels of these nine key genes in IL‐1β‐induced C28/i2 cells, and the expression of *CDKN2A* and *FDX1* were also elevated in conditioned monocytes, while the expression of *GLS* and *MTF1* were significantly decreased. In vitro experiments demonstrated that the expression levels of these 7/10 CRGs were significantly increased in chondrocytes induced by IL‐1β, and upon stimulation with cuproptosis inducers, chondrocyte apoptosis was exacerbated, accompanied by an increase in the expression of cuproptosis‐related proteins. These further substantiated our research findings and indicated that the nine selected cuproptosis genes have high potential for application in the diagnosis of OA.

## 1 INTRODUCTION

Osteoarthritis (OA) is the most prevalent form of arthritis, characterized by articular cartilage degeneration, hyperosteogeny at joint margins, subchondral bone loss, synovial inflammation and increased blood vessels.^1^, ^2^ According to the World Health Organization statistics, approximately 10%–15% of the global population aged 45 above is affected by this disease. With the deepening of global population aging, the incidence of this disease continues to climb. Currently, over 500 million individuals worldwide have been diagnosed with OA.^3^, ^4^ Given the higher personal medical expenditures and substantial economic burden faced by patients with osteoarthritic conditions due to decreased productivity, in‐depth research into its pathomechanisms and potential biomarkers holds significant clinical and socio‐economic importance. OA is considered a multifactorial pathogenic process, with its susceptibility potentially influenced by genetic factors, endocrine dysregulation, age, gender, non‐physiological mechanical loads, nutritional status, among multiple others.^5^, ^6^, ^7^, ^8^ Joint tissues adjust the trace element content during the lengthy physiological remodelling process to adapt to external changes. Hence, any deviation in the levels of trace elements within the joint, whether excessive or deficient, could potentially compromise joint function and increase the risk of developing osteoarthritic conditions.^9^ For instance, trace elements such as copper play a positive role in the maintenance of bones and joints at certain concentrations, but levels that are either too low or too high may induce the onset of osteoarthritis.^10^, ^11^

Copper is an essential trace element in the human body, playing a crucial role as a catalytic cofactor in various biological processes, including mitochondrial respiration, antioxidant defence and the synthesis of biological compounds.^12^, ^13^ The homeostasis of intracellular copper is meticulously regulated through processes of absorption, distribution and elimination. However, excessive accumulation of copper within cells can lead to cytotoxicity and cell death.^14^ The impact of copper on cartilage is bidirectional. Copper serves as a cofactor for several enzymes in cartilage, including superoxide dismutase, cytochrome C, ascorbate oxidase and lysyl oxidase. Particularly, lysyl oxidase, a copper‐dependent amine oxidase, is a key enzyme in the cross‐linking of collagen and elastin, further promoting the formation of cartilage.^15^, ^16^ Previous studies have indicated that copper deficiency impairs the activity of lysyl oxidase, disrupts collagen‐elastin cross‐linking, resulting in incomplete cartilage and increased risk of OA.^17^, ^18^ Excessive copper ions induce a plethora of oxidative reactions, causing joint damage.^19^ Furthermore, epidemiological studies have identified a significant association between copper metabolism disorder and the incidence of OA, although the molecular mechanism remains underlying this relationship remain unclear.^18^

Cuproptosis, a distinct form of cell death, sets itself apart from other forms such as apoptosis, ferroptosis and necrosis. It occurs when excessive intracellular copper ion accumulation leads to abnormal build‐up of lipoylated proteins in the mitochondria, disrupting the mitochondrial respiration‐associated iron–sulfur cluster proteins, and causing a protein toxicity stress response that culminates in cell death.^20^ In OA joints, the mitochondrial function of chondrocytes and synovial cells is severely disordered, characterized by enhanced inflammation, increased apoptosis, heightened catabolic metabolism and reduced mitochondrial biogenesis.^21^, ^22^ Yazar et al. found that the concentration of Cu and Fe in the synovial fluid of OA patients was higher than that in healthy individuals (*p* < 0.05).^23^ Additionally, cell death is closely associated with synovitis in OA, as seen in fibroblasts and mononuclear cells.^24^, ^25^ Based on this, we hypothesize that cuproptosis is involved in the pathogenesis and progression of osteoarthritis. Currently, the potential regulatory mechanisms of cuproptosis in OA remain unclarified and warrant further exploration. The goal is that genes related to cuproptosis may become targets for the treatment of OA.

In this study, we systematically analysed the differential expression of Cuproptosis‐related genes (CRGs) and their immunological characteristics among healthy subjects, OA patients and rheumatoid arthritis (RA) patients. Firstly, we obtained the microarray datasets GSE12021, GSE55235 and GSE55457 as the analysis datasets, determined the expression profiles of CRGs, and conducted functional relevance analyses, including correlation analysis, clinical prediction model construction, subgroup identification and Gene Set Enrichment Analysis (GSEA). Secondly, we explored the abundance of 22 types of immune cells and their correlation with CRGs expression based on the datasets. Additionally, the expression levels of CRGs in chondrocytes and monocytes under inflammation induction were validated by real‐time quantitative polymerase chain reaction (RT‐PCR) technology. Further experimental studies elucidated the effects of cuproptosis inducers on chondrocytes, aiming to confirm the potential of CRGs as clinical diagnostic biomarkers for OA and to explore their feasibility as targets for pharmacological intervention.

## 2 MATERIALS AND METHODS

### 2.1 Data acquisition and study design

We obtained chip data and clinical information of synovial samples from patients with OA and normal controls from the GEO database. Specifically, we downloaded the data with accession numbers GSE12021, GSE55235,^26^ and GSE55457.^27^ The samples included in our study were sourced from Homo sapiens.

The dataset GSE12021 includes a total of 30 samples, comprising of 8 normal samples, 10 samples from OA patients and 12 samples from RA patients. Similarly, the dataset GSE55235 consists of 30 samples, with 10 normal samples, 10 samples from OA patients and 10 samples from RA patients. Lastly, the dataset GSE55457 includes 33 samples, with 10 normal samples, 10 samples from OA patients and 13 samples from RA patients. The specific sample groups selected for analysis will be further described in the subsequent text (Table 1).

**TABLE 1:** GEO datasets.

	GSE12021	GSE55235	GSE55457
Organism	Homo sapiens	Homo sapiens	Homo sapiens
Experiment type	Expression profiling by array	Expression profiling by array	Expression profiling by array
Platforms	GPL96	GPL96	GPL96
Sample(number)			
normal	8	10	10
OA	10	10	10
RA	12	10	13
Total	30	30	33

**FIGURE 1:**
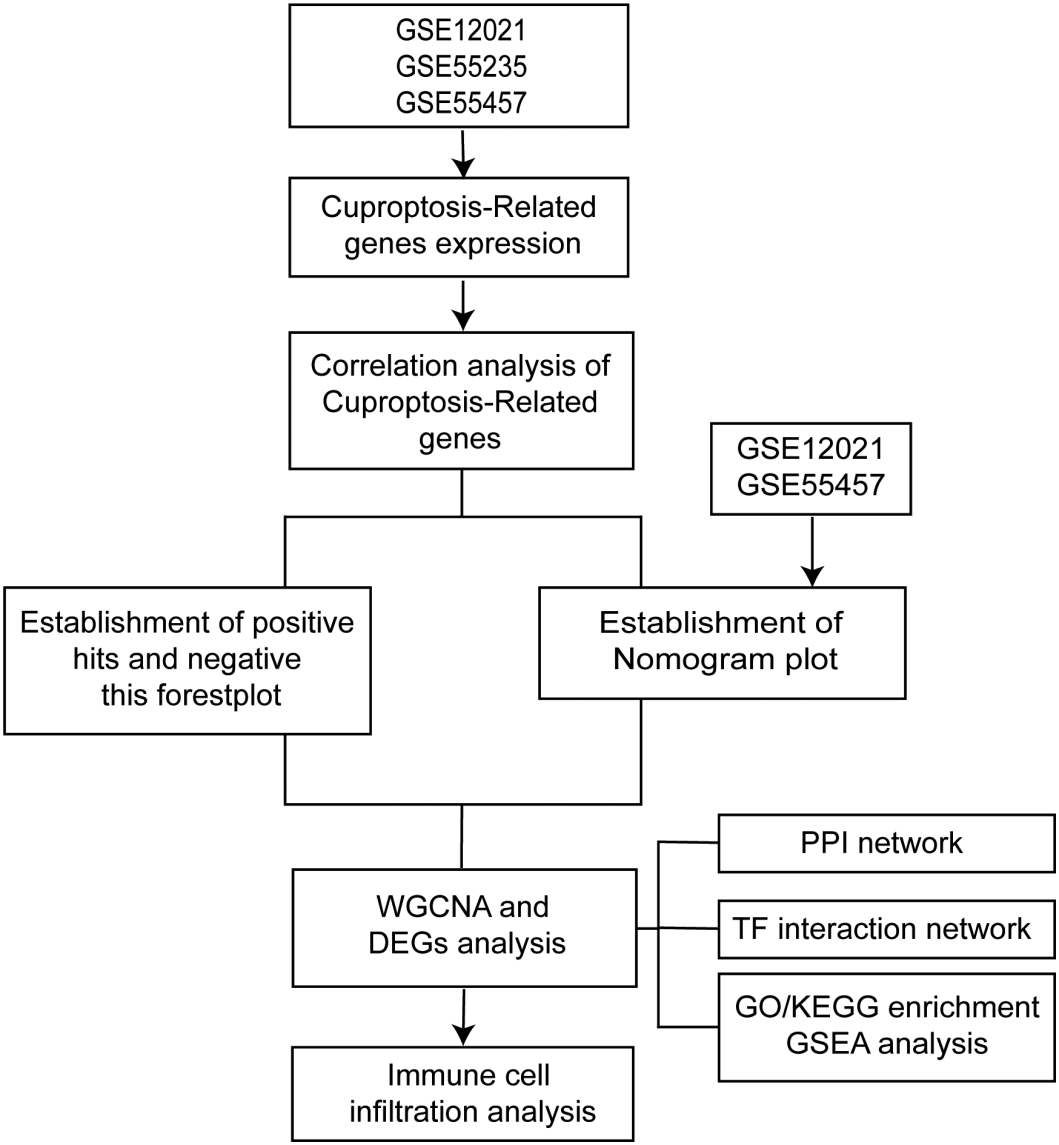
Chart of Overall Project Analysis.

We utilized the R package affy^28^ to import the CEL files from three datasets and conducted RMA background correction and quantiles normalization. Afterwards, we employed the normalizeBetweenArrays function from the limma^29^ package to harmonize the data across the three datasets. To address batch effects, we applied the removeBatchEffect function. Lastly, we merged the data from the GSE12021 and GSE55457 datasets and generated a Nomogram plot. Also, the forest plots of the positive hits and negative hits were established. Then, use WGCNA to analyse differentially expressed genes between normal samples and patients, identify multiple modules and extract key module hub genes. Establishment of protein–protein interaction network for hub genes, GO/KEGG enrichment analysis, GSEA analysis and immune infiltration analysis (Figure 1).

### 2.2 Expression and correlation analysis of CRGs in OA

Based on previous literature reports, we have identified 10 genes associated with cuproptosis (*CDKN2A*, *FDX1*, *DLD*, *DLAT*, *LIAS*, *GLS*, *LIPT1*, *MTF1*, *PDHA1* and *PDHB*).^20^, ^30^ In order to assess their expression patterns, we compared the expression levels of these 10 genes between normal samples and patients with OA or RA using a combined dataset (GSE12021, GSE55235 and GSE55457). We then generated a boxplot visualization of the expression data using the ggpubr package. Then, we downloaded genome annotation information from GENECODE,^31^ obtained full length information of hg38.chrom.sizes from UCSC,^32^ and the protein–protein interaction network (PPI) were constructed in the STRING^33^ database (https://string‐db.org/) with the above 10 regulatory factors as inputs. Visualization was performed using Cytoscape software. Spearman correlation analysis was performed to integrate the dataset (GSE12021, GSE55235 and GSE55457) by examining the correlation between CRGs categorized as positive hits and negative hits. To visualize the results, a scatter plot was created using the ggpubr package^34^ to depict the significantly correlated genes with *FDX1* (a key regulatory gene in cuproptosis), and 2 genes (*LIPT1* and *PDHB*) with differential expression between normal and OA conditions.

### 2.3 Clinical risk analysis and evaluation of CRGs in OA

We use the RIdeogram^35^ and ggbio^36^ packages, draw positive hits of cuproptosis (*FDX1*, *DLD*, *DLAT*, *LIAS*, *LIPT1*, *PDHA1*, *PDHB*) against negative hits of cuproptosis (*CDKN2A*, *GLS*, *MTF1*).^20^, ^30^ Through logistic regression analysis, we explored the association between 10 CRGs and OA and RA, and plotted a forest plot to display the odds ratios and *P*‐value for each gene. We integrated the GSE12021 and GSE55457 datasets, constructed nomograms for positive hits and negative hits, and utilized the integrated data (GSE12021, GSE55457 and GSE55235) to assess the model's efficacy through calibration curves.

### 2.4 Molecular typing analysis was performed according to hub gene

The differentially expressed genes (13,515 genes) that have been selected will be subjected to variance analysis in order to calculate their expression levels. Genes with a variance that exceeds all quartiles (6751 genes) will be further utilized for constructing a co‐expression network. Subsequently, the differentially expressed genes will undergo WGCNA analysis,^37^ which involves several steps such as soft thresholding, network construction, hierarchical clustering, computation of module correlations, construction of visual gene networks, and correlation analysis with phenotype data. The purpose of this process is to identify modules that encompass genes related to cuproptosis by maximizing correlation. The top three modules will be identified, and the genes located within these modules will be extracted. By utilizing the STRING^33^ database with these genes as input, PPI will be obtained. According to the official manual of WGCNA, the relationship between genes and modules will be evaluated using KME calculation. Typically, a threshold of |KME| ≥ 0.8 is commonly used to select hub genes.

### 2.5 Differential gene expression analysis and GO/KEGG, GSEA enrichment analysis

We performed differential gene expression analysis on integrated datasets (GSE12021, GSE55235 and GSE55457) using the limma package and generated a volcano plot using the ggplot2 package.^38^ The differentially expressed genes identified with the limma package were then subjected to GO and KEGG enrichment analysis (Table 2).

**TABLE 2:** GO/KEGG enrichment analysis results.

GO:						
ONTOLOGY	ID	Description	*p*‐value	*p* adjust	*q*‐value	Count
BP	GO:1990868	Response to chemokine	6.15E‐10	6.15E‐10	7.15E‐07	11
BP	GO:1990869	Cellular response to chemokine	6.15E‐10	6.15E‐10	7.15E‐07	11
BP	GO:0050864	Regulation of B cell activation	1.07E‐09	1.07E‐09	8.31E‐07	14
BP	GO:0032496	Response to lipopolysaccharide	2.67E‐09	2.67E‐09	1.55E‐06	17
BP	GO:0070098	Chemokine‐mediated signalling pathway	3.72E‐09	3.72E‐09	1.73E‐06	10
BP	GO:0097529	Myeloid leukocyte migration	5.20E‐09	5.20E‐09	1.89E‐06	14
BP	GO:0050871	Positive regulation of B cell activation	5.77E‐09	5.77E‐09	1.89E‐06	12
BP	GO:0002237	Response to molecule of bacterial origin	6.51E‐09	6.51E‐09	1.89E‐06	17
BP	GO:0002685	Regulation of leukocyte migration	2.18E‐08	2.18E‐08	5.64E‐06	13
BP	GO:0045444	Fat cell differentiation	5.30E‐08	5.30E‐08	1.20E‐05	13
BP	GO:0002696	Positive regulation of leukocyte activation	5.67E‐08	5.67E‐08	1.20E‐05	17
BP	GO:0031960	Response to corticosteroid	7.17E‐08	7.17E‐08	1.39E‐05	11
BP	GO:0050867	Positive regulation of cell activation	8.37E‐08	8.37E‐08	1.50E‐05	17
CC	GO:0072562	Blood microparticle	5.41E‐09	5.41E‐09	9.90E‐07	12
CC	GO:0042571	Immunoglobulin complex, circulating	2.15E‐08	2.15E‐08	1.97E‐06	9
CC	GO:0019814	Immunoglobulin complex	1.99E‐06	1.99E‐06	0.000122	10
CC	GO:0062023	Collagen‐containing extracellular matrix	1.97E‐05	1.97E‐05	0.000902	14
CC	GO:0009897	External side of plasma membrane	0.000206	0.000206	0.007535	12
MF	GO:0005539	Glycosaminoglycan binding	8.71E‐09	8.71E‐09	2.50E‐06	14
MF	GO:0003823	Antigen binding	1.61E‐08	1.61E‐08	2.50E‐06	12
MF	GO:0034987	Immunoglobulin receptor binding	2.17E‐08	2.17E‐08	2.50E‐06	9
MF	GO:0008009	Chemokine activity	1.72E‐07	1.72E‐07	1.49E‐05	7
MF	GO:0008201	Heparin binding	1.34E‐06	1.34E‐06	9.28E‐05	10
MF	GO:0042379	Chemokine receptor binding	1.87E‐06	1.87E‐06	0.000108	7
MF	GO:0048018	Receptor ligand activity	3.74E‐06	3.74E‐06	0.000185	16
MF	GO:0030546	Signalling receptor activator activity	4.38E‐06	4.38E‐06	0.000189	16
MF	GO:0005125	Cytokine activity	5.19E‐06	5.19E‐06	0.000189	11
MF	GO:0001228	DNA‐binding transcription activator activity, RNA polymerase II‐specific	5.46E‐06	5.46E‐06	0.000189	15
MF	GO:0001216	DNA‐binding transcription activator activity	6.09E‐06	6.09E‐06	0.000192	15
MF	GO:0001664	G protein‐coupled receptor binding	3.58E‐05	3.58E‐05	0.001033	11
MF	GO:1901681	Sulfur compound binding	7.48E‐05	7.48E‐05	0.001994	10
MF	GO:0071889	14‐3‐3 protein binding	0.000129	0.000129	0.0032	4
MF	GO:0042834	Peptidoglycan binding	0.00044	0.00044	0.009531	3
MF	GO:0045236	CXCR chemokine receptor binding	0.00044	0.00044	0.009531	3

KEGG:					
ID	Description	*p*‐value	*p* adjust	*q*‐value	Count
hsa05323	Rheumatoid arthritis	2.09E‐14	2.09E‐14	2.47E‐12	15
hsa05417	Lipid and atherosclerosis	4.69E‐12	4.69E‐12	2.77E‐10	18
hsa04657	IL‐17 signalling pathway	1.01E‐11	1.01E‐11	3.97E‐10	13
hsa04668	TNF signalling pathway	1.40E‐09	1.40E‐09	4.14E‐08	12
hsa04064	NF‐kappa B signalling pathway	1.03E‐07	1.03E‐07	2.13E‐06	10
hsa05134	Legionellosis	1.08E‐07	1.08E‐07	2.13E‐06	8
hsa05167	Kaposi sarcoma‐associated herpesvirus infection	6.70E‐07	6.70E‐07	1.13E‐05	12
hsa04061	Viral protein interaction with cytokine and cytokine receptor	8.28E‐07	8.28E‐07	1.22E‐05	9
hsa04620	Toll‐like receptor signalling pathway	1.16E‐06	1.16E‐06	1.51E‐05	9
hsa05202	Transcriptional misregulation in cancer	4.53E‐06	4.53E‐06	5.34E‐05	11
hsa04380	Osteoclast differentiation	6.55E‐06	6.55E‐06	6.90E‐05	9
hsa05169	Epstein–Barr virus infection	7.02E‐06	7.02E‐06	6.90E‐05	11
hsa05142	Chagas disease	9.85E‐06	9.85E‐06	8.93E‐05	8
hsa05144	Malaria	1.18E‐05	1.18E‐05	9.93E‐05	6
hsa05162	Measles	1.28E‐05	1.28E‐05	0.000101	9
hsa05166	Human T‐cell leukaemia virus 1 infection	1.72E‐05	1.72E‐05	0.000127	11
hsa05163	Human cytomegalovirus infection	1.95E‐05	1.95E‐05	0.000135	11
hsa04010	MAPK signalling pathway	4.78E‐05	4.78E‐05	0.000307	12
hsa04060	Cytokine‐cytokine receptor interaction	4.94E‐05	4.94E‐05	0.000307	12
hsa05219	Bladder cancer	6.09E‐05	6.09E‐05	0.000359	5
hsa04933	AGE‐RAGE signalling pathway in diabetic complications	7.60E‐05	7.60E‐05	0.000427	7
hsa04659	Th17 cell differentiation	0.000124	0.000124	0.000664	7
hsa05140	Leishmaniasis	0.000141	0.000141	0.00072	6
hsa04062	Chemokine signalling pathway	0.000162	0.000162	0.000794	9
hsa05210	Colorectal cancer	0.000259	0.000259	0.00122	6
hsa05222	Small cell lung cancer	0.000373	0.000373	0.001693	6
hsa04068	FoxO signalling pathway	0.000409	0.000409	0.001786	7
hsa04213	Longevity regulating pathway—multiple species	0.000443	0.000443	0.001864	5
hsa05216	Thyroid cancer	0.000558	0.000558	0.002268	4

GO enrichment analysis is a commonly used method for studying the functional enrichment of genes at different levels and dimensions. This analysis typically encompasses three levels: biological process (BP), molecular function (MF) and cellular component (CC). KEGG, a widely‐used database, contains information related to genomes, pathways, diseases and drugs. In this study, the R package ClusterProfiler is employed to annotate all significantly differentially expressed genes with GO terms, with the goal of identifying significantly enriched BP. The results of the enrichment analysis are then visualized using the R packages GOplot and topGO. The significance threshold for the enrichment analysis is set at *p* < 0.05.

Gene Set Enrichment Analysis (GSEA) is a computational method utilized to assess whether a predefined set of genes exhibits statistically significant differences between two biological states (Table 3). This method is commonly employed to estimate alterations in pathway and biological process activities in a given dataset of gene expression. To investigate the disparities in biological processes between two groups of samples, we retrieved reference gene sets ‘c5.go.v7.4.entrez.gmt’ and ‘c2.cp.kegg.v7.4. entrez.gmt’ from the MSigDB database based on the differentially expressed genes identified in the differential expression analysis conducted in section 1.4 (*p* < 0.05, |log2FC|>0). We conducted enrichment analysis and visualization of the dataset using the GSEA method implemented in the R package ‘clusterProfiler’. *p* < 0.05 was considered significant.

**TABLE 3:** GSEA analysis result.

GO:					
ONTOLOGY	ID	Description	NES	*p*‐value	*p* adjust
BP	GO:1901360	Organic cyclic compound metabolic process	−3.13155	1.05E‐08	5.52E‐06
BP	GO:0006725	Cellular aromatic compound metabolic process	−2.94935	2.50E‐07	6.57E‐05
BP	GO:0046483	Heterocycle metabolic process	−2.84337	1.15E‐06	0.000202
BP	GO:0006139	Nucleobase‐containing compound metabolic process	−2.65489	1.70E‐06	0.000223
CC	GO:0031974	Membrane‐enclosed lumen	−2.59692	3.09E‐06	0.000253
CC	GO:0043233	Organelle lumen	−2.59692	3.09E‐06	0.000253
BP	GO:0044237	Cellular metabolic process	−2.67803	3.37E‐06	0.000253
BP	GO:0006807	Nitrogen compound metabolic process	−2.62935	3.94E‐06	0.000259
BP	GO:0044271	Cellular nitrogen compound biosynthetic process	−2.60989	1.00E‐05	0.000528
BP	GO:0034641	Cellular nitrogen compound metabolic process	−2.70899	1.00E‐05	0.000528
BP	GO:0044260	Cellular macromolecule metabolic process	−2.5622	1.33E‐05	0.000632
BP	GO:1901362	Organic cyclic compound biosynthetic process	−2.55474	1.44E‐05	0.000632
CC	GO:0070013	Intracellular organelle lumen	−2.52707	2.12E‐05	0.000856
CC	GO:0043231	Intracellular membrane‐bounded organelle	−2.51626	2.32E‐05	0.000871
BP	GO:0009059	Macromolecule biosynthetic process	−2.40197	2.55E‐05	0.000891
BP	GO:0033554	Cellular response to stress	−2.46313	2.71E‐05	0.000891
BP	GO:0090304	Nucleic acid metabolic process	−2.46504	3.17E‐05	0.000933
BP	GO:0034645	Cellular macromolecule biosynthetic process	−2.53585	3.57E‐05	0.000933
BP	GO:0031326	Regulation of cellular biosynthetic process	−2.46504	3.88E‐05	0.000933
BP	GO:0044238	Primary metabolic process	−2.42894	4.09E‐05	0.000933
BP	GO:0019219	Regulation of nucleobase‐containing compound metabolic process	−2.47753	4.18E‐05	0.000933
MF	GO:0097159	Organic cyclic compound binding	−2.44193	4.38E‐05	0.000933
CC	GO:0005622	Intracellular anatomical structure	−2.49585	4.75E‐05	0.000933
BP	GO:0010556	Regulation of macromolecule biosynthetic process	−2.42679	5.13E‐05	0.000933
BP	GO:2000112	Regulation of cellular macromolecule biosynthetic process	−2.42679	5.13E‐05	0.000933
BP	GO:0018130	Heterocycle biosynthetic process	−2.46822	5.24E‐05	0.000933
BP	GO:0019438	Aromatic compound biosynthetic process	−2.46822	5.24E‐05	0.000933
BP	GO:0034654	Nucleobase‐containing compound biosynthetic process	−2.46822	5.24E‐05	0.000933
BP	GO:0008152	Metabolic process	−2.42808	5.27E‐05	0.000933
CC	GO:0005634	Nucleus	−2.4335	5.47E‐05	0.000933
BP	GO:0071704	Organic substance metabolic process	−2.44375	5.50E‐05	0.000933
CC	GO:0043227	Membrane‐bounded organelle	−2.36775	6.32E‐05	0.001039
BP	GO:0045934	Negative regulation of nucleobase‐containing compound metabolic process	−2.4105	6.97E‐05	0.00111
BP	GO:0044249	Cellular biosynthetic process	−2.48464	7.19E‐05	0.001113
BP	GO:0009058	Biosynthetic process	−2.4106	8.00E‐05	0.001202
BP	GO:0016070	RNA metabolic process	−2.4134	8.69E‐05	0.001265
BP	GO:0009889	Regulation of biosynthetic process	−2.3815	8.90E‐05	0.001265
CC	GO:0043229	Intracellular organelle	−2.33187	9.42E‐05	0.001305
BP	GO:1901576	Organic substance biosynthetic process	−2.3624	0.000101	0.001369
MF	GO:1901363	Heterocyclic compound binding	−2.34974	0.000157	0.00206
BP	GO:0051252	Regulation of RNA metabolic process	−2.33294	0.000165	0.002116
BP	GO:0051172	Negative regulation of nitrogen compound metabolic process	−2.32391	0.000175	0.002137
BP	GO:0009890	Negative regulation of biosynthetic process	−2.32509	0.000177	0.002137
BP	GO:1901564	Organonitrogen compound metabolic process	−2.27337	0.000179	0.002137
BP	GO:0006357	Regulation of transcription by RNA polymerase II	−2.35189	0.000189	0.002166
BP	GO:0006366	Transcription by RNA polymerase II	−2.35189	0.000189	0.002166
BP	GO:0051173	Positive regulation of nitrogen compound metabolic process	−2.32858	0.000213	0.002379
CC	GO:0005829	Cytosol	−2.30825	0.000235	0.002578
BP	GO:0031323	Regulation of cellular metabolic process	−2.19967	0.000265	0.00285
CC	GO:0031981	Nuclear lumen	−2.2318	0.000284	0.002984
BP	GO:0051254	Positive regulation of RNA metabolic process	−2.23423	0.00031	0.003175
BP	GO:0045944	Positive regulation of transcription by RNA polymerase II	−2.30605	0.000314	0.003175
BP	GO:0045935	Positive regulation of nucleobase‐containing compound metabolic process	−2.23633	0.000328	0.003253
BP	GO:0080090	Regulation of primary metabolic process	−2.23551	0.000351	0.003418
CC	GO:0005654	Nucleoplasm	−2.15514	0.000363	0.003475
BP	GO:0009987	Cellular process	−2.16377	0.00039	0.003665
BP	GO:0010558	Negative regulation of macromolecule biosynthetic process	−2.23689	0.000408	0.003702
BP	GO:2000113	Negative regulation of cellular macromolecule biosynthetic process	−2.23689	0.000408	0.003702
BP	GO:0051171	Regulation of nitrogen compound metabolic process	−2.35349	0.000418	0.003722
BP	GO:0031325	Positive regulation of cellular metabolic process	−2.24676	0.000455	0.00373
CC	GO:0043226	Organelle	−2.17956	0.000463	0.00373
BP	GO:0006351	Transcription, DNA‐templated	−2.283	0.000475	0.00373
BP	GO:0006355	Regulation of transcription, DNA‐templated	−2.283	0.000475	0.00373
BP	GO:0032774	RNA biosynthetic process	−2.283	0.000475	0.00373
BP	GO:0097659	Nucleic acid‐templated transcription	−2.283	0.000475	0.00373
BP	GO:1903506	Regulation of nucleic acid‐templated transcription	−2.283	0.000475	0.00373
BP	GO:2001141	Regulation of RNA biosynthetic process	−2.283	0.000475	0.00373
BP	GO:0010605	Negative regulation of macromolecule metabolic process	−2.18138	0.000518	0.004003
BP	GO:0031327	Negative regulation of cellular biosynthetic process	−2.21122	0.000554	0.004225
BP	GO:0045893	Positive regulation of transcription, DNA‐templated	−2.2132	0.000597	0.004365
BP	GO:1902680	Positive regulation of RNA biosynthetic process	−2.2132	0.000597	0.004365
BP	GO:1903508	Positive regulation of nucleic acid‐templated transcription	−2.2132	0.000597	0.004365
BP	GO:0035295	Tube development	−2.19593	0.000647	0.00466
MF	GO:0005488	Binding	−2.11519	0.000672	0.004777
BP	GO:0009968	Negative regulation of signal transduction	−2.14145	0.00078	0.005471
BP	GO:0010557	Positive regulation of macromolecule biosynthetic process	−2.13519	0.000873	0.006041
BP	GO:0072359	Circulatory system development	−2.0779	0.000896	0.006119
BP	GO:0009888	Tissue development	−2.05091	0.000918	0.006142
BP	GO:0043170	Macromolecule metabolic process	−2.0831	0.000922	0.006142
BP	GO:0031328	Positive regulation of cellular biosynthetic process	−2.15833	0.001157	0.007606
BP	GO:0051253	Negative regulation of RNA metabolic process	−2.13002	0.001219	0.007915
BP	GO:0010468	Regulation of gene expression	−2.14531	0.001295	0.008305
BP	GO:0010604	Positive regulation of macromolecule metabolic process	−2.03835	0.001442	0.009112
BP	GO:0010648	Negative regulation of cell communication	−2.10243	0.001473	0.009112
BP	GO:0023057	Negative regulation of signalling	−2.10243	0.001473	0.009112
BP	GO:0009891	Positive regulation of biosynthetic process	−2.0847	0.00149	0.009112
BP	GO:0060429	Epithelium development	−2.12013	0.001584	0.009575
BP	GO:0045892	Negative regulation of transcription, DNA‐templated	−2.01377	0.001937	0.011322
BP	GO:1902679	Negative regulation of RNA biosynthetic process	−2.01377	0.001937	0.011322
BP	GO:1903507	Negative regulation of nucleic acid‐templated transcription	−2.01377	0.001937	0.011322
MF	GO:0003677	DNA binding	−2.00814	0.002046	0.011736
BP	GO:0070482	Response to oxygen levels	−2.00269	0.002053	0.011736
BP	GO:0009893	Positive regulation of metabolic process	−2.08368	0.00208	0.011764
BP	GO:0035239	Tube morphogenesis	−2.04452	0.00214	0.011976
BP	GO:0060255	Regulation of macromolecule metabolic process	−2.0157	0.002226	0.012327
BP	GO:0009892	Negative regulation of metabolic process	−2.03776	0.002293	0.01248
BP	GO:0001568	Blood vessel development	−2.02693	0.002353	0.01248
BP	GO:0001944	Vasculature development	−2.02693	0.002353	0.01248
BP	GO:0044281	Small molecule metabolic process	−1.99851	0.002375	0.01248
BP	GO:0050896	Response to stimulus	−1.94401	0.002398	0.01248
BP	GO:0006979	Response to oxidative stress	−1.98346	0.002403	0.01248
BP	GO:0031324	Negative regulation of cellular metabolic process	−2.06075	0.002431	0.01248
BP	GO:0048585	Negative regulation of response to stimulus	−2.02319	0.002444	0.01248
CC	GO:0005737	Cytoplasm	−1.98418	0.002651	0.013302
BP	GO:0035556	Intracellular signal transduction	−1.95547	0.002655	0.013302
BP	GO:1901566	Organonitrogen compound biosynthetic process	−1.96206	0.002974	0.01476
CC	GO:0000785	Chromatin	−2.00921	0.003097	0.015223
CC	GO:0005575	Cellular_component	−1.90841	0.003209	0.015419
CC	GO:0110165	Cellular anatomical entity	−1.90841	0.003209	0.015419
BP	GO:0048646	Anatomical structure formation involved in morphogenesis	−1.96351	0.003224	0.015419
BP	GO:0071310	Cellular response to organic substance	−1.95188	0.003329	0.015774
BP	GO:0048514	Blood vessel morphogenesis	−1.94614	0.003391	0.015928
BP	GO:0048523	Negative regulation of cellular process	−1.86814	0.003422	0.015928
BP	GO:0008150	Biological_process	−1.89256	0.003514	0.016214
BP	GO:0006464	Cellular protein modification process	−1.8667	0.004118	0.018674
BP	GO:0036211	Protein modification process	−1.8667	0.004118	0.018674
BP	GO:0051384	Response to glucocorticoid	−1.98027	0.00419	0.018836
BP	GO:0009611	Response to wounding	−1.89998	0.004568	0.020363
MF	GO:0003674	Molecular_function	−1.87901	0.004772	0.021093
BP	GO:0019222	Regulation of metabolic process	−1.91067	0.00482	0.021126
BP	GO:0010467	Gene expression	−1.97874	0.005015	0.021683
MF	GO:0003824	Catalytic activity	−1.92336	0.005029	0.021683
MF	GO:0036094	Small molecule binding	−1.86909	0.005405	0.023114

BP	GO:0048513	Animal organ development	−1.87512	0.005477	0.023233
CC	GO:0071944	Cell periphery	1.822593	0.00571	0.024028
CC	GO:0043228	Non‐membrane‐bounded organelle	−1.94728	0.006009	0.024865
CC	GO:0043232	Intracellular non‐membrane‐bounded organelle	−1.94728	0.006009	0.024865
BP	GO:0001525	Angiogenesis	−1.87238	0.006051	0.024865
BP	GO:0009966	Regulation of signal transduction	−1.87978	0.006155	0.025042
CC	GO:0005694	Chromosome	−1.93121	0.006254	0.025042
BP	GO:0009653	Anatomical structure morphogenesis	−1.87495	0.00627	0.025042
BP	GO:0043085	Positive regulation of catalytic activity	−1.90855	0.006284	0.025042
BP	GO:0051338	Regulation of transferase activity	−1.89867	0.006528	0.025819
BP	GO:0042981	Regulation of apoptotic process	−1.79781	0.006634	0.025846
BP	GO:0043067	Regulation of programmed cell death	−1.79781	0.006634	0.025846
BP	GO:0043408	Regulation of MAPK cascade	−1.95456	0.006885	0.02663
BP	GO:0000122	Negative regulation of transcription by RNA polymerase II	−1.94822	0.007134	0.027349
MF	GO:0003676	Nucleic acid binding	−1.89761	0.007175	0.027349
BP	GO:0043068	Positive regulation of programmed cell death	−1.87726	0.007531	0.028498
BP	GO:0006915	Apoptotic process	−1.78996	0.007863	0.029083
BP	GO:0012501	Programmed cell death	−1.78996	0.007863	0.029083
BP	GO:0001666	Response to hypoxia	−1.88063	0.007906	0.029083
BP	GO:0036293	Response to decreased oxygen levels	−1.88063	0.007906	0.029083
BP	GO:0043412	Macromolecule modification	−1.77634	0.008238	0.029883
BP	GO:1901700	Response to oxygen‐containing compound	−1.77615	0.008238	0.029883
BP	GO:0042221	Response to chemical	−1.87517	0.008417	0.030324
BP	GO:0030154	Cell differentiation	−1.8097	0.009164	0.032569
BP	GO:0048869	Cellular developmental process	−1.8097	0.009164	0.032569
BP	GO:0009790	Embryo development	−1.86139	0.009496	0.033522
BP	GO:0044267	Cellular protein metabolic process	−1.80288	0.010161	0.035631
BP	GO:0010941	Regulation of cell death	−1.75675	0.010542	0.036653
BP	GO:0048519	Negative regulation of biological process	−1.78584	0.010707	0.036653
MF	GO:0001216	DNA‐binding transcription activator activity	−1.90306	0.010731	0.036653
MF	GO:0001228	DNA‐binding transcription activator activity, RNA polymerase II‐specific	−1.90306	0.010731	0.036653
BP	GO:0034097	Response to cytokine	−1.70526	0.011447	0.038847
BP	GO:0005975	Carbohydrate metabolic process	−1.76179	0.012277	0.041133
BP	GO:1902532	Negative regulation of intracellular signal transduction	−1.82691	0.012277	0.041133
BP	GO:0070887	Cellular response to chemical stimulus	−1.85711	0.012885	0.042831
MF	GO:0004888	Transmembrane signalling receptor activity	1.863314	0.01311	0.042831
MF	GO:0038023	Signalling receptor activity	1.863314	0.01311	0.042831
MF	GO:0060089	Molecular transducer activity	1.863314	0.01311	0.042831
BP	GO:0051726	Regulation of cell cycle	−1.83626	0.014079	0.045714
BP	GO:0043065	Positive regulation of apoptotic process	−1.71921	0.014243	0.045961
BP	GO:0006950	Response to stress	−1.81	0.014599	0.046823
MF	GO:0000976	Transcription regulatory region sequence‐specific DNA binding	−1.78894	0.015455	0.047821
MF	GO:0000987	Cis‐regulatory region sequence‐specific DNA binding	−1.78894	0.015455	0.047821
MF	GO:0001067	Regulatory region nucleic acid binding	−1.78894	0.015455	0.047821
MF	GO:0003690	Double‐stranded DNA binding	−1.78894	0.015455	0.047821
MF	GO:0043565	Sequence‐specific DNA binding	−1.78894	0.015455	0.047821
MF	GO:1990837	Sequence‐specific double‐stranded DNA binding	−1.78894	0.015455	0.047821
BP	GO:0019538	Protein metabolic process	−1.78613	0.015861	0.048788

### 2.6 Correlation analysis of immune subtypes

Twenty‐eight immune cells were collected.^39^ The expression profile data of OA patients were extracted from the integrated dataset (GSE12021, GSE55235 and GSE55457). To analyse this data, we utilized the GSVA package^40^ to perform single‐sample gene set enrichment analysis (ssGSEA). This allowed us to compare the activity of different gene sets in each patient. Next, we used the ConsensuClusterPlus package^41^ in R to cluster the ssGSEA results and determine the optimal number of clusters based on the expression of 10 CRGs. As a result, the ssGSEA results were divided into two distinct clusters. We then calculated the differential expression genes, totaling 810 genes, between these two clusters. Furthermore, we compared and visualized the abundance of immune cells using boxplots.

We performed correlation analyses to examine the relationship between the expression of genes associated with cuproptosis and immune subtypes as well as the correlation between different levels of immune cell infiltration. Combinations with a significance level of *p* < 0.01 and a correlation coefficient >0.7 were selected for the analysis. Scatter plots were generated to visually represent the results of the correlation analyses.

### 2.7 Cell culture

The human C28/i2 chondrocytes were derived from a human juvenile costal cartilage^42^ and were provided by Dr. Mary Goldring from Cornell University. The chondrocytes grown in DMEM/F12 supplement with 10% (v/v) fetal bovine serum (FBS) and 100 units/mL penicillin–streptomycin at 37°C in a 5% CO_2_ incubator. The THP‐1 monocyte cells grown in RPMI‐1640 supplement with 10% (v/v) FBS and 100 units/mL penicillin–streptomycin at 37°C in a 5% CO_2_ incubator.

### 2.8 RT‐PCR

The C28/i2 chondrocytes were cultured in serum‐supplemented DMEM/F12 with or without 10 ng/mL IL‐1β for 48 h, the cells were washed twice with 1 × PBS. Then, the total RNA from C28/i2 cells by Trizol. mRNA was reverse‐transcribed into cDNA using HiScript II Q RT SuperMix. The relative mRNA expression levels of *CDKN2A*, *DLAT*, *DLD*, *FDX1*, *LIAS*, *LIPT1*, *MTF1*, *PDHB* in C28/i2 chondrocytes were determined by using qPCR with the cDNA template and ChamQ SYBR qPCR master mix in a CFX96 Touch qPCR System (BioRad, Hercules, CA, USA). The sequences of the forward and reverse primers used in this study is listed in Table 4. The data were normalized to GAPDH and analysed via 2^−ΔΔCt^ method (A relative quantification strategy, which is a method to analyse the relative changes in gene expression from real‐time quantitative PCR experiments).

The THP‐1 cells were cultured in conditioned medium for 48 h. This conditional medium refers to C28/i2 cell medium with or without 10 ng/mL IL‐1β. The specific steps: The C28/i2 chondrocytes were cultured in serum‐supplemented DMEM/F12 with or without 10 ng/mL IL‐1β for 24 h, the cells were washed twice with 1× PBS and subsequently cultured in serum‐supplemented normal medium, and conditioned medium (CM) was harvested after 24 h. Then, we use the CM to culture THP‐1 cells for 48 h for RT‐PCR. The total RNA from THP‐1 cells was extracted by Trizol. mRNA was reverse‐transcribed into cDNA using HiScript II Q RT SuperMix. The relative mRNA expression levels of *CDKN2A*, *FDX1*, *GLS*, *MITF1* in the THP‐1 monocyte cells were determined by using qPCR with the cDNA template and ChamQ SYBR qPCR master mix in a CFX96 Touch qPCR System (BioRad, Hercules, CA, USA). The sequences of the forward and reverse primers used in this study is listed in Table 4. The data were normalized to GAPDH and analysed via 2^−ΔΔCt^ method.

**TABLE 4:** Primers employed in this study.

Gene symbol	Forward primer (5′‐3′)	Reverse primer (5′‐3′)
*PDHB*	ATGCTCCTGCTGTTCGTGTC	TTGCAGTACAAATCCAGGTGC
*MTF1*	GAGAGACTATCAGATCCAATCAG	GGTGTTTTCTTTCTGGCTCC
*GLS*	AGCACTCAAATCTACAGGATTGC	CACAAATCGGGACTGAATTTGG
*LIAS*	TGGTGTGACTACTTCAGAACCT	GGAATAGGGCATGTGGATTTAGCA
*DLD*	CCGAACTGATGTAAGTAAACGGTC	GCCCACGTATTTGAGTTCCGTA
*CDKN2A*	CCACCCCGCTTTCGTAGTT	AGTGAAAAGGCAGAAGCGGT
*LIPT1*	TTCTGAACTGAATCTCGCTCTGTTG	GAGGCAGGAGAATCGCTTGAAC
*FDX1*	AGTTGGTGATTCTCTGCTAGATGTTG	AAGATGAGGTGACAGGTTGAACAAG

### 2.9 CCK‐8 assay

C28/i2 cells were prepared as cell suspensions at a density of 2 × 10^4^ cells/mL. Subsequently, 200 μL of the cell suspension was added to each well of a 96‐well plate. After the cells adhered to the bottom of the wells, they were treated with Elesclomol (ES, STA‐4783, MCE) at 10^−10^ M, 10^−9^ M, 10^−8^ M, 10^−7^ M, 10^−6^ M, 10^−5^ M concentration with or without 1 μM CuCl_2_ (C3279, sigma) or 10 μM Tetrathiomolybdate (TTM, HY‐128530, MCE) or 1 mM GSH (G6013, sigma) for 24, 48 and 72 h. Following removal of the culture medium, 100 μL of cell culture medium containing 10% CCK‐8 was added to each well. The plate was then incubated at 37°C for 2 h, and the optical density (OD) was measured at 450 nm.

### 2.10 Cell apoptosis

C28/i2 cells were prepared as cell suspensions at a density of 7.5 × 10^4^ cells/mL. Subsequently, 2 mL of the cell suspension was added to each well of a 6‐well plate. After the cells adhered to the bottom of the wells, they were treated with serum‐supplemented medium with or without 10^−8^ M ES/10^−8^ M ES + 1 μM CuCl_2_/10^−8^ M ES + 1 μM CuCl_2_ + 10 μM TTM/10^−8^ M ES + 1 μM CuCl_2_ + 1 mM GSH for 48 h. Subsequently, the cells were digested using trypsin and collected through centrifugation. They were washed once with 1× PBS. The cells were stained with 5 μL annexin V‐FITC in the absence of light for 20 min, followed by another round of centrifugation and 1× PBS wash once. Lastly, the cells were stained with 5 μL propidium iodide (PI) in the absence of light for 5 min. The apoptotic cells were identified using a flow cytometer.

### 2.11 Western blot

C28/i2 cells were seeded in 6‐well plate at a density of 7.5 × 10^4^ cells/mL and treated with or without 10 ng/mL IL‐1β or 10^−8^ M ES/10^−8^ M ES + 1 μM CuCl_2_/10^−8^ M ES + 1 μM CuCl_2_ + 10 μM TTM/10^−8^ M ES + 1 μM CuCl_2_ + 1 mM GSH. After treatment, cells were washed with pre‐cooled 1× PBS and lysed with RIPA containing protease inhibitor and phosphatase inhibitor. Proteins of equal quality were separated by SDS‐PAGE and then transferred onto a PVDF membrane. The membrane was sealed with 5% skimmed milk powder, then the primary antibody of FDX1 (1:1000, A20895, Abclone) was bound target protein at 4°C overnight, and the specific secondary antibody was incubated at room temperature for 2 h, and the immunoreactive band was detected by ECL and quantified using Image J software.

### 2.12 Statistical analysis

Data calculation and statistical analysis for bioinformatics analysis were conducted using R programming (version 4.0.2) (https://www.r‐project.org/). For the comparison of two groups with continuous variables, the statistical significance of normally distributed variables was assessed using the independent Student's *t*‐test, while the difference between non‐normally distributed variables was analysed using the Mann–Whitney *U* test (also known as the Wilcoxon rank‐sum test). All statistical *p*‐value were two‐tailed, and a *p*‐value less than 0.05 was considered statistically significant.

Data analysis was performed using GraphPad Prism software. Student's *t*‐test or one‐way ANOVA was used to analyse the significant difference in groups. Data are presented as the means ± standard error of the mean (SEM). In all cases, *p* < 0.05 was considered statistically significant.

## 3 RESULTS

### 3.1 Expression and correlation analysis of CRGs in OA and RA

**FIGURE 2:**
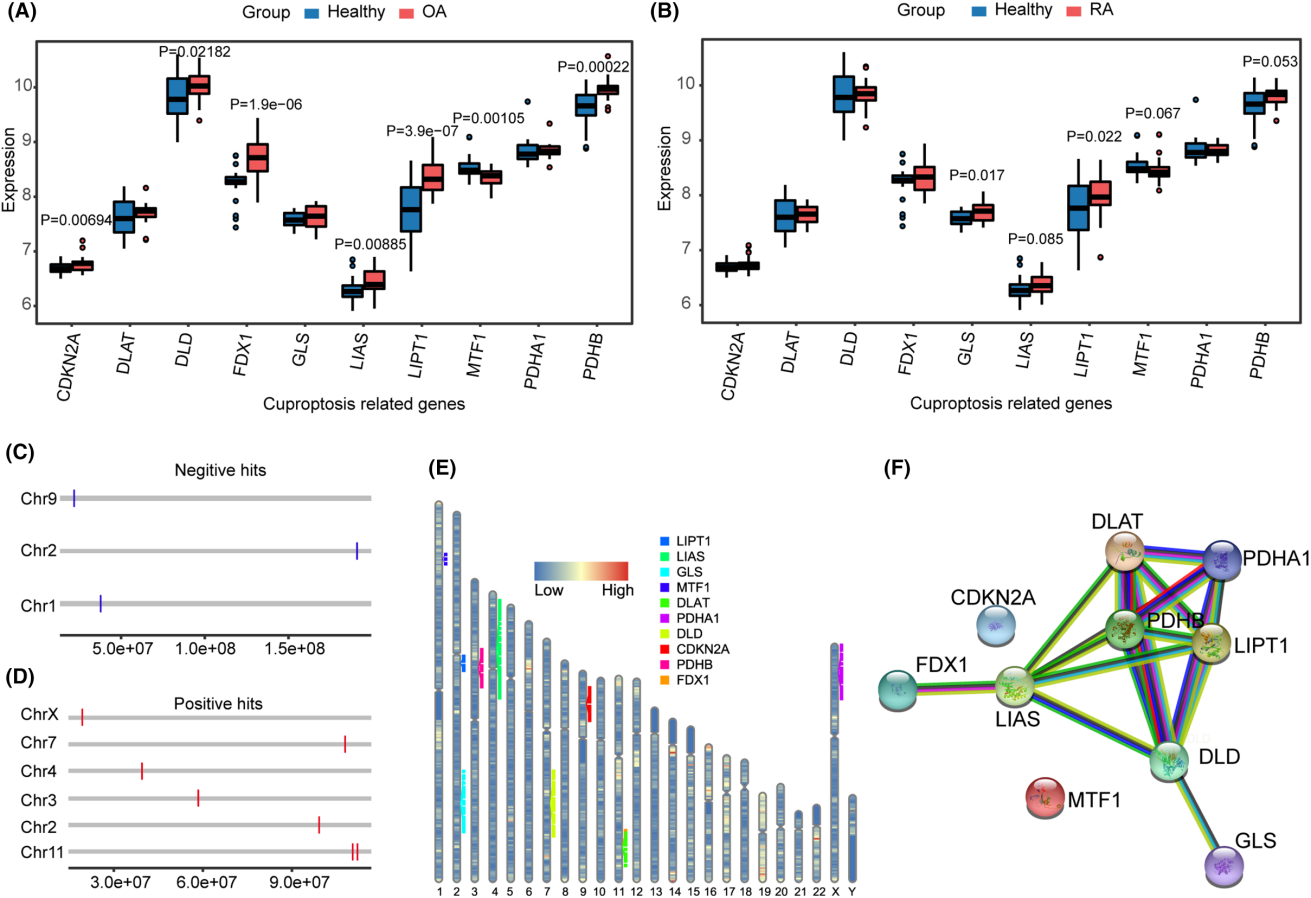
Expression analysis of cuproptosis‐related genes in OA. (A) Box plots of gene expression in normal samples and OA samples for 10 cuproptosis‐related genes. (B) Box plots of gene expression in normal samples and RA samples for 10 cuproptosis‐related gene. The figure displays all *P*‐value in numerical format, with significance denoted as *p* < 0.05. Normal samples are represented in blue, while OA or RA samples are depicted in pink. (C) Location of Negative hits in genes associated with copper death on chromosomes. (D) Location of positive hits in genes associated with copper death on chromosomes. (E) Localization of 10 cuproptosis‐related genes on 24 chromosomes. (F) Protein interaction network for 10 cuproptosis‐related genes.

The heat map in Figure 2A shows the distribution of 10 CRGs in OA and RA. When OA patients were compared with normal samples, there were significant differences in seven out of 10 genes, except for *DLAT*, *GLS* and *PDHA1*. Among the genes with significant differences, the expression levels of *CDKN2A*, *DLD*, *FDX1*, *LIAS*, *LIPT1* and *PDHB* were significantly increased, while the expression level of *MTF1* was decreased (Figure 2A). When RA patients were compared with normal samples, there were significant increase in 2 out of 10 genes, including *GLS* and *LIPT1* (Figure 2B). The positions of 10 CRGs on chromosomes were all plotted in Figure 2C–E. Figure 2C,D showed the chromosomal positions of positive hits and negative hits, respectively. Figure 2E showed a panorama of CRGs expression in 22 autochromosomes and 2 sex chromosomes, whose PPI networks were plotted in Figure 2F.

**FIGURE 3:**
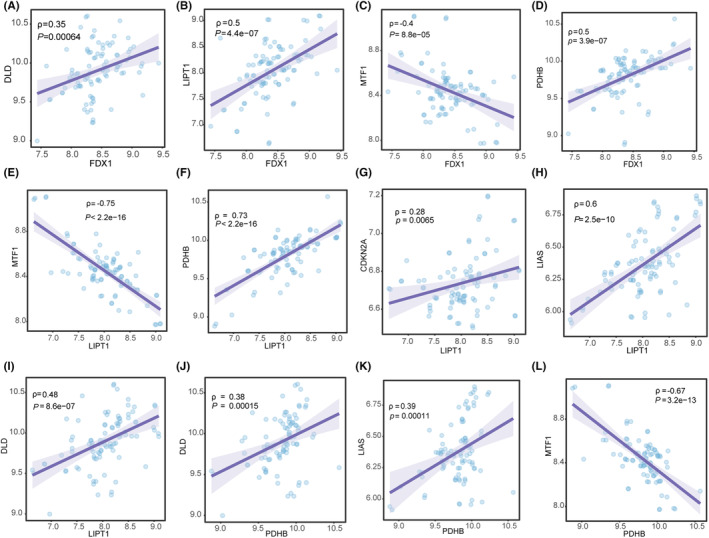
Correlation analysis of cuproptosis‐related genes belonging to positive hits and negative hits in the integration of the dataset. Correlation Scatter Plots for *FDX1*: (A) *FDX1*‐*DLD*, (B) *FDX1*‐*LIPT1*, (C) *FDX1*‐*MTF1*, (D) *FDX1*‐*PDHB*. Correlation Scatter Plots with *LIPT1*: (E) *LIPT1*‐*MTF1*, (F) *LIPT1*‐*PDHB*, (G) *LIPT1*‐*CDKN2A*, (H) *LIPT1*‐*LIAS*, (I) *LIPT1*‐*DLD*. Correlation Scatter Plots with *PDHB*: (J) *PDHB*‐*DLD*, (K) *PDHB*‐*LIAS*, (L) *PDHB*‐*MTF1*.

In the integration of the dataset, a Spearman correlation analysis was performed on cuproptosis‐related genes belonging to positive hits and negative hits. Scatterplots were generated for combinations significantly correlated with the cuproptosis‐associated key regulatory genes *FDX1* and two genes (*LIPT1*, *PDHB*) with expression differences in all samples of the datasets. The combinations with *P*‐value less than 0.05 are shown in Figure 3 A‐L. Among them, *FDX1*‐*DLD*, *FDX1*‐*LIPT1*, *LIPT1*‐*PDHB*, *LIPT1*‐*CDKN2A*, *LIPT1*‐*LIAS*, *LIPT1*‐*DLD*, *PDHB*‐*DLD* and *PDHB*‐*LIAS* show positive correlation (Figure 3A,B,D,F,G–L), among them, *FDX1*‐*LIPT1*, *FDX1‐PDHB*, *LIPT1*‐*PDHB* and *LIPT1*‐*LIAS* had moderate positive correlations (0.5≤*r*<0.7). While *FDX1*‐*MTF1*, *LIPT1*‐*MTF1* and *PDHB*‐*MTF1* show negative correlation (Figure 3C,E,L), among them, *PDHB* had moderate positive correlations with *MTF1* (−0.7<*r*≤−0.5).

### 3.2 Clinical risk assessment of CRGs in OA

**FIGURE 4:**
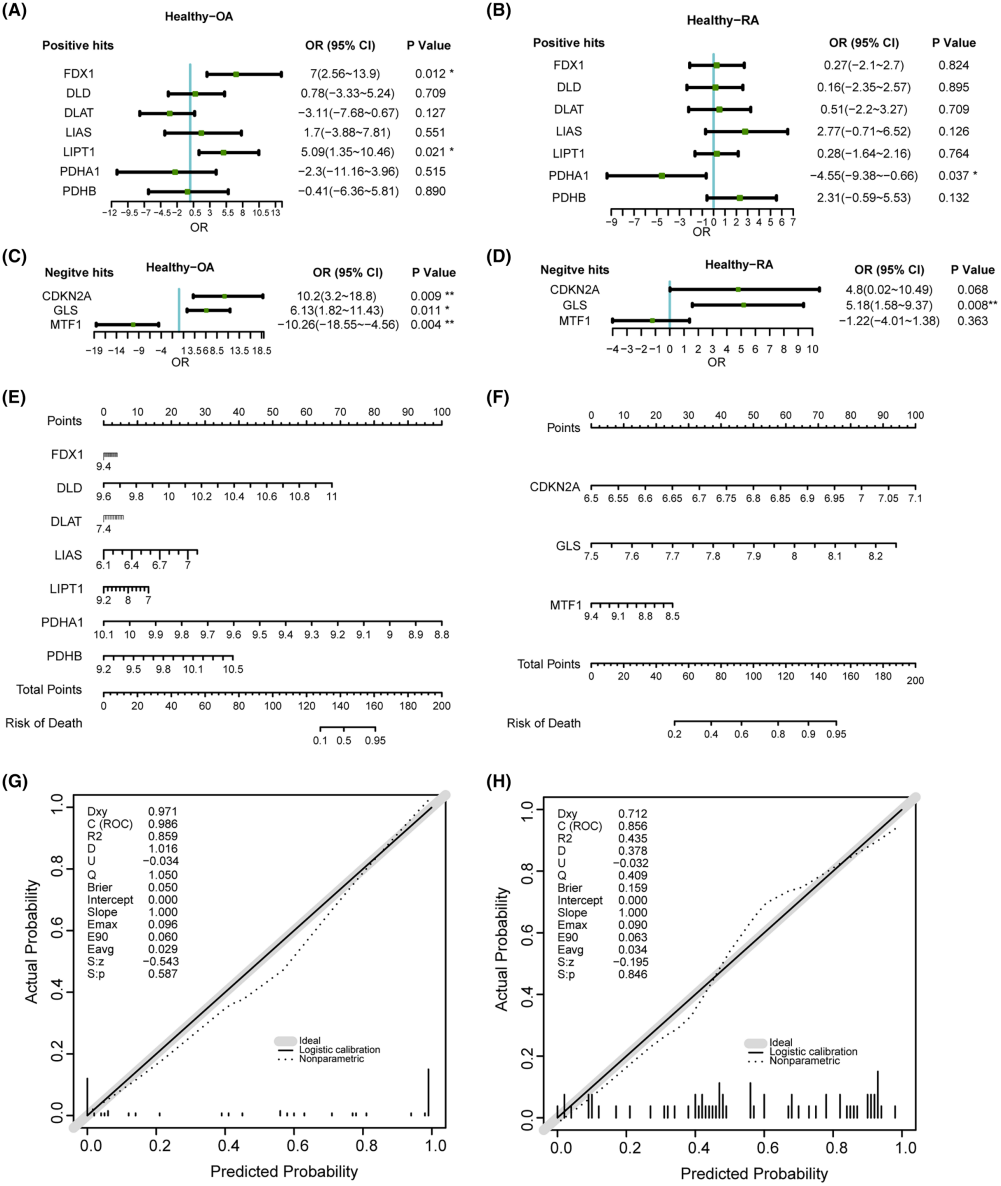
Logistic regression analysis and clinical prediction of cuproptosis‐related genes in OA and RA. (A) Forest map of positive hits in cuproptosis‐related genes of normal samples compared with OA group. (B) Forest map of positive hits in cuproptosis‐related genes of normal samples compared with RA group. (C) Forest map of negative hits in cuproptosis‐related genes of normal samples compared with OA group. (D) Forest map of negative hits in cuproptosis‐related genes of normal samples compared with RA group. The nomogram of positive hits (E) and negative hits (F) of cuproptosis‐related genes (GSE12021 and GSE55457). Correction curves for positive hits (G) and negative hits (H) of cuproptosis‐related genes (GSE55235). Dxy represents the correlation between predicted values and actual values. The C (ROC) index, also known as the area under the ROC curve, is calculated using the formula C = (1 + Dxy) / 2. R^2^ denotes the coefficient of determination for the model. D represents the discrimination index. U signifies the unreliability index, where a smaller value indicates a better predictive model. Q represents the quality index, where a higher value indicates a better predictive model; The Brier score represents the mean squared error between predicted values and actual values, with a smaller Brier score indicating better calibration. Emax indicates the maximum absolute difference between predicted values and actual values, with a smaller Emax indicating a model closer to the ideal state. Eavg represents the average difference between predicted values and actual values. E90 represents the 90th percentile of the differences between predicted values and actual values.

Logistic regression was employed to investigate the correlation between 10 CRGs and OA or RA. Forest plots were constructed to present the findings. In the group of CRGs displaying positive associations, *FDX1* and *LIPT1* were identified as statistically significant factors in relation to OA (*p* < 0.05, Figure 4A), while *PDHA1* was identified as statistically significant factors in relation to RA (*p* < 0.05, Figure 4B). Likewise, within the group of CRGs showing negative associations, *CDKN2A*, *GLS* and *MTF1* were found to have significant connections with OA (*p* < 0.05, Figure 4C), while *GLS* was found to have significant connections with RA (*p* < 0.05, Figure 4D).

In the predictive model for risk of death in OA constructed using the GSE12021 and GSE55457 datasets, a nomogram incorporating positive hits and negative hits (Figure 4E,F) was employed to visually analyse the interrelationships among model variables. The predictive performance of the model was evaluated using an area under the Receiver Operating Characteristic (ROC) curve derived from the dataset for OA application (GSE12021, GSE55457 and GSE55235) (Figure 4G,H). Dxy represents the correlation between predicted values and actual values, with values of 0.971 (positive hits) and 0.712 (negative hits) in this context, indicating a good correlation. The C (ROC) index, also known as the area under the ROC curve, is calculated using the formula C = (1 + Dxy)/2, yielding values of 0.986 (positive hits) and 0.856 (negative hits) here, demonstrating the model's superior discriminative ability. *R* ^2^ denotes the coefficient of determination for the model. D represents the discrimination index, where a higher value indicates a better predictive model, with values of 1.016 (positive hits) and 0.378 (negative hits) here, suggesting a higher discrimination index for the positive hits predictive model. *U* signifies the unreliability index, where a smaller value indicates a better predictive model, with values of −0.034 (positive hits) and − 0.032 (negative hits) here. *Q* represents the quality index, where a higher value indicates a better predictive model, with values of 1.050 (positive hits) and 0.409 (negative hits) here. The Brier score represents the mean squared error between predicted values and actual values, with a smaller Brier score indicating better calibration, showing values of 0.050 (positive hits) and 0.159 (negative hits) here. The intercept represents the model's intercept, and the slope represents the model's slope. Emax indicates the maximum absolute difference between predicted values and actual values, with a smaller Emax indicating a model closer to the ideal state, showing values of 0.096 (positive hits) and 0.09 (negative hits) here. Eavg represents the average difference between predicted values and actual values, showing values of 0.029 (positive hits) and 0.034 (negative hits) here. E90 represents the 90th percentile of the differences between predicted values and actual values, showing values of 0.060 (positive hits) and 0.063 (negative hits) here. $:*z* and $:*p* represent the *z*‐value and *p*‐value for the *z*‐test. With resulting *p‐*value of 0.587 (positive hits) and 0.846 (negative hits), both greater than 0.05, confirm the model's reliability. Comprehensive analysis of various indicators suggests that this predictive model exhibits high predictive accuracy, with the positive hits predictive model being particularly outstanding.

### 3.3 Molecular typing according to hub gene in OA

**FIGURE 5:**
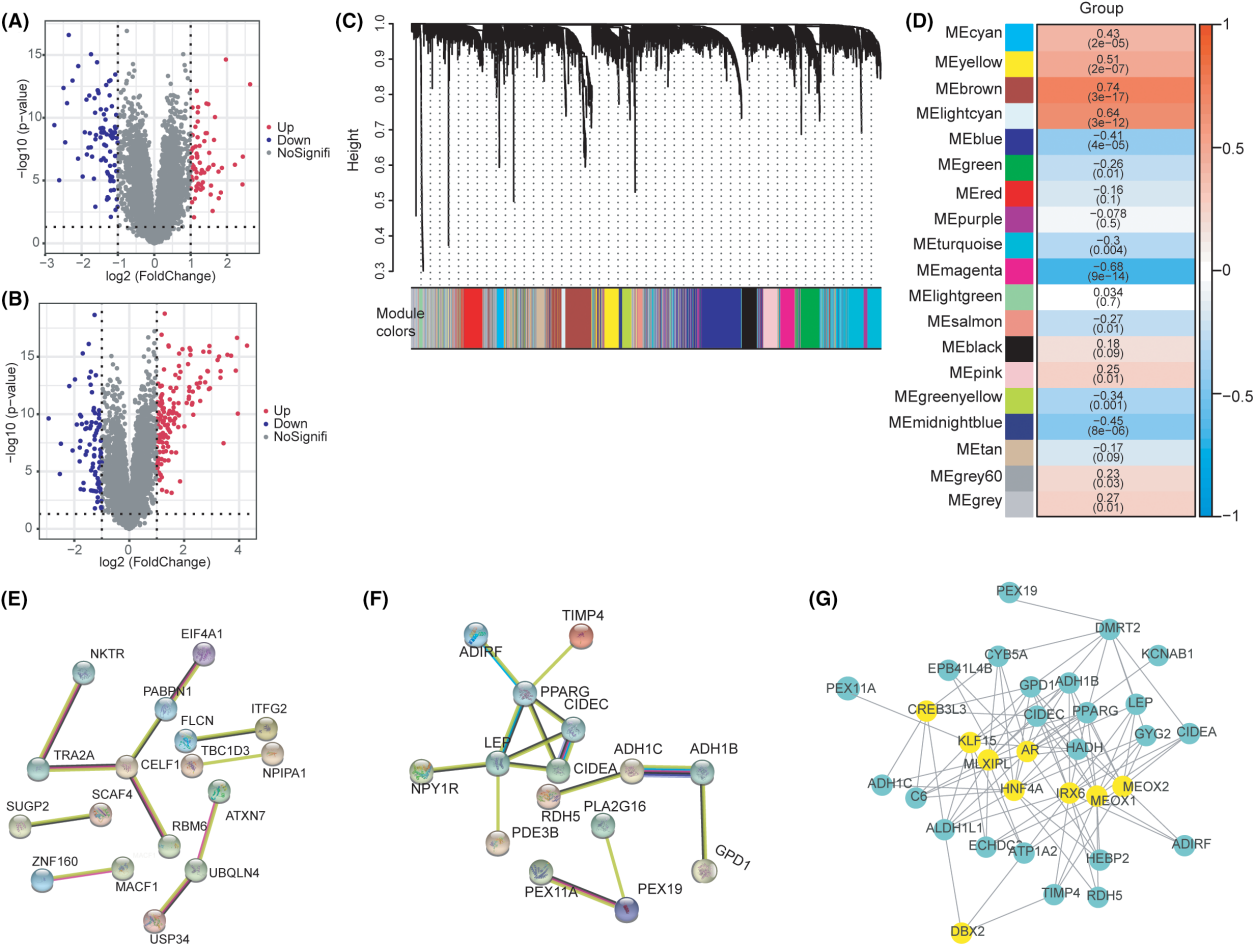
Identification of OA phenotypic related genes and prediction of hub gene transcription factors. (A) Volcano plot displaying the differentially expressed genes in normal samples versus OA. (B) Volcano plot displaying the differentially expressed genes in normal samples vs RA. The *x*‐axis represents log2(Foldchange), and the *y*‐axis represents −log10 (*p*‐value). Each gene is represented by a dot, with red indicating up‐regulated genes, blue indicating down‐regulated genes, and grey indicating undifferentially expressed genes. (C) Gene Tree map obtained by average linkage hierarchical clustering, with colour rows representing 19 module assignments determined by Dynamic Tree Cut. (D) A heat map of the association of 19 module characteristic genes with disease.The redder the correlation coefficient is closer to 1, the bluer the correlation coefficient is closer to −1, the different colour blocks on the left represent different modules, the numbers outside the heat map parentheses represent the correlation coefficient, and the numbers inside the parentheses represent the significance *p*‐value. (E) PPI network diagram of hub gene in green module. (F) PPI network diagram of hub gene in greenyellow module. (G) Transcription factor interaction network diagram of greenyellow module and green module gene. Green represents hub gene, yellow represents transcription factor.

The differential gene expression (DGE) was analysed in the three datasets (GSE12021, GSE5523526 and GSE55457) There were 68 up‐regulated genes and 93 down‐regulated genes in healthy samples and OA samples (Figure 5A). There were 150 up‐regulated genes and 84 down‐regulated genes in the healthy samples and the RA samples (Figure 5B). Differential genes were employed for WGCNA analysis. The soft threshold was set to 0.85, and the module with the greatest number of genes associated with cuproptosis was identified. The genes within this module were extracted. The modules yielded key genes in quantities of 64, 21 and 26, respectively, resulting in a total of 111 hub genes being extracted (Figure 5C,D). We employed KME ≥0.8 for gene network prediction of the interplay within the cuproptosis‐related gene module (Figure 5E,F). For transcription factor prediction, the top 10 transcription factors identified were *AR*, *CREB3L3*, *DBX2*, *DMRT2*, *HNF4A*, *IRX6*, *KLF15*, *MEOX1*, *MEOX2*, *MLXIPL* (Figure 5G).

### 3.4 Gene differential expression analysis and GO/KEGG, GSEA enrichment analysis

**FIGURE 6:**
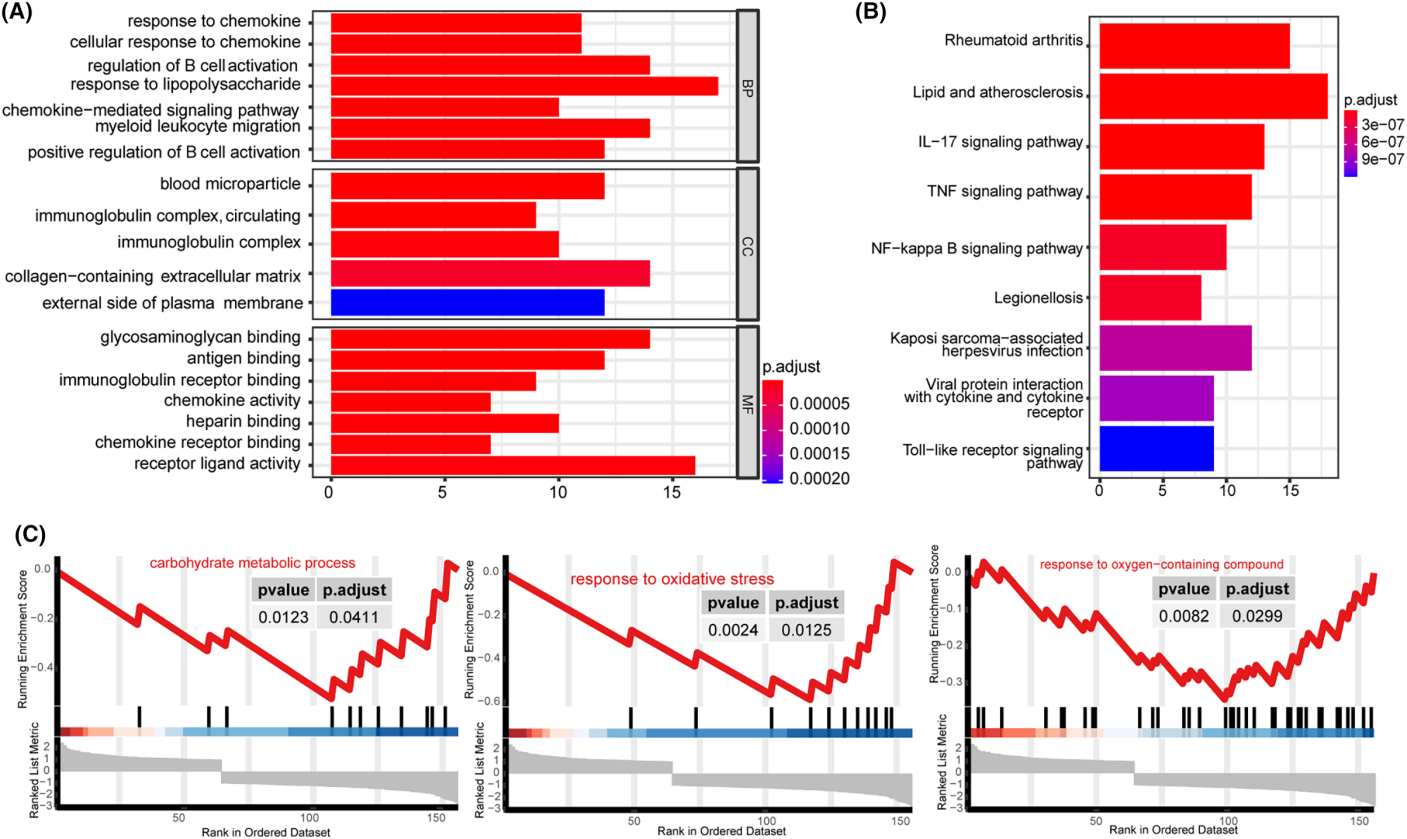
GO/KEGG, GSEA enrichment analysis. (A) Bar chart illustrating the enrichment results for Gene Ontology (GO). (B) Bar chart illustrating the enrichment results for Kyoto Encyclopedia of Genes and Genomes (KEGG). The *x*‐axis represents −log10 (*p*‐value), the *y*‐axis represents the enriched GO terms and pathways, and the colour of the bars represents the corrected *p*‐value. (C) GSEA Mountain plot for GO enrichment analysis: Response to oxidative stress, carbohydrate metabolic process and response to oxygen‐containing compound. The *x*‐axis represents the rank of genes in the differentially expressed gene list, where up‐regulated genes have positive ranks (>0) and down‐regulated genes have negative ranks (<0). The upper *y*‐axis represents the enrichment fraction, while the lower *y*‐axis represents the logFC (log Fold Change) value.

To investigate the biological processes or functions influenced by the differentially expressed genes (DEGs) in the all normal‐OA comparison of these datasets (GSE12021, GSE55235 26 and GSE55457), we performed GO, KEGG enrichment and GSEA enrichment analyses on all 810 DEGs. The GO enrichment analysis revealed a total of 513 enriched GO terms, which encompassed categories for BP, MF and CC. For visual representation, a bar chart depicting the top 20 terms with the lowest *p*‐value was generated. Notably, the BP category among the top‐ranking terms included response to chemokine, cellular response to chemokine and regulation of B cell activation; the MF category among the top‐ranking terms included glycosaminoglycan binding, antigen binding and immunoglobulin receptor binding; the CC category among the top‐ranking terms included blood microparticle, immunoglobulin complex, circulating and immunoglobulin complex (Figure 6A).

KEGG enrichment analysis was performed on 80 KEGG pathways, with a significance level of *p* < 0.05. Figure 6B displays the bar plot and bubble plot representing the top 9 pathways, ranked according to their *p*‐value. The pathways included in the plot are labelled as rheumatoid arthritis, Lipid and atherosclerosis, IL‐17 signalling pathway, TNF signalling pathway and other pathways are related to rheumatology and immunology (Figure 6B).

In the GSEA analysis, to enhance the enrichment of pathways, we modified the threshold criteria for the |log2FC|>0. Figure 6C displays the resulting pathways enriched in GO related to cuproptosis, which include carbohydrate metabolic process, response to oxidative stress and response to oxygen‐containing compounds.

### 3.5 Unsupervised clustering analysis of CRGs and correlation analysis of immune feature subtypes

**FIGURE 7:**
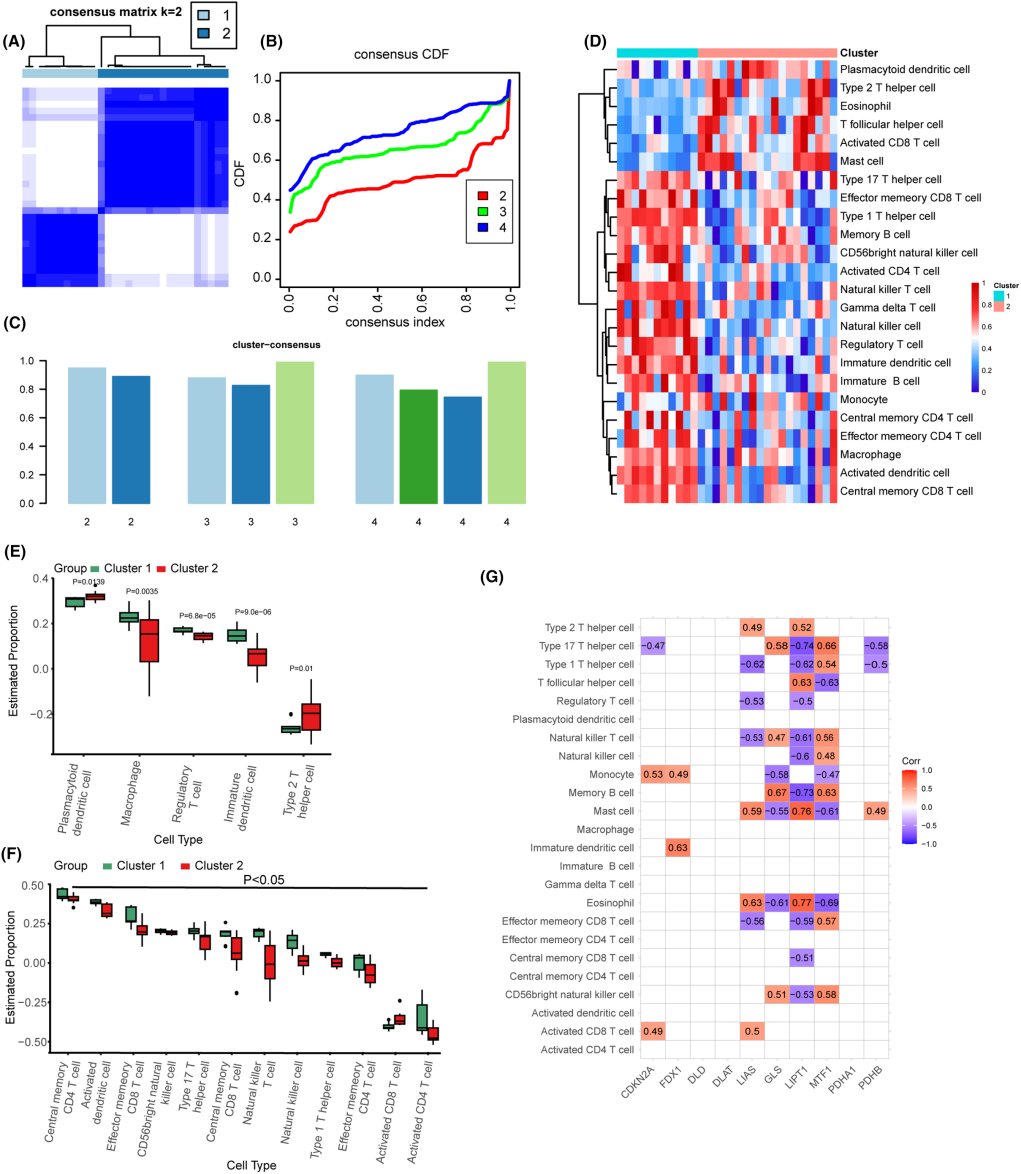
Unsupervised cluster analysis of genes related to cuproptosis and correlation analysis of immune characteristic subtypes. (A) Heatmap displaying the proportional matrix of OA sample co‐occurrences in the integrated dataset. (B) Cumulative Distribution Function (CDF) plot of consistent clustering for *k* = 2–4 in the OA group of the integrated dataset. (C) Histogram depicting the results of ICL clustering, with each colour representing a specific cluster. (D) Heatmap showcasing the results of ssGSEA analysis on immune cells and hub genes. (E) Box plot comparing the proportion of proto‐cancer immune cells between the two clusters (*p*‐value indicated in the figure). (F) Box plot comparing the proportion of cancer‐suppressing immune cells between the two clusters (*p*‐value indicated in the results section). (G) Heat map showcases the correlation analysis between the proportion of 24 immune cell types and the expression levels of 10 genes associated with cuproptosis. Notably, significant correlations are highlighted using distinct colours, with correlation coefficients close to −1 depicted as shades of purple, and correlation coefficients close to 1 depicted as shades of red.

To investigate the modification patterns of cuproptosis in OA, we extracted expression profile data of OA patients from the integrated dataset. We then conducted single sample gene set enrichment analysis (ssGSEA) to analyse the expression profiles. Subsequently, unsupervised consensus clustering analysis was performed based on 10 CRGs (Figure 7A–C), resulting in the identification of two patient clusters. We compared the abundance of immune cells between these two clusters and visualized the clustering expression heatmap in Figure 7D. The box plots in Figure 7E,F demonstrate that the estimated proportions of immune cells (Type 2 T helper cell, Eosinophil, T follicular helper cell, Activated CD8 T cell, Mast cell) were lower in cluster 1 and higher in cluster 2. The difference in proportions between cancerous cells (Plasmacytoid dendritic cell, Macrophage, Regulatory T cell, Immature dendritic cell, Type 2 T helper c, *p* = 0.0139, *p* = 0.0035, *p* = 6.8e‐05, *p* = 9.0e‐06, *p* = 0.01, Figure 7E) and anticancer cells (Activated CD4 T cell, Activated CD8 T cell, Central memory CD4 T cell, Central memory CD8 T cell, Effector memeory CD4 T cell, Effector memeory CD8 T cell, Type 1 T helper cell, Type 17 T helper cell, Activated dendritic cell, CD56 bright natural killer cell, Natural killer cell, Natural killer T cell)^43^ is deemed statistically significant (*p* = 0.02709, *p* = 3.9e−06, *p* = 0.00065, *p* = 0.04752, *p* = 0.00821, *p* = 0.00041, *p* = 5.8e−06, *p* = 6.9e−06, *p* = 1.5e−06, *p* = 0.01116, *p* = 0.00064, *p* = 0.01323, Figure 7F). Furthermore, the correlation of immune cell and CRGs was analysed by using ssGSEA method. All CRGs exhibiting significant correlations are represented by colours in Figure 7G.

### 3.6 The expression of CGRs in chondrocyte and monocyte under IL‐1β stimulation

**FIGURE 8:**
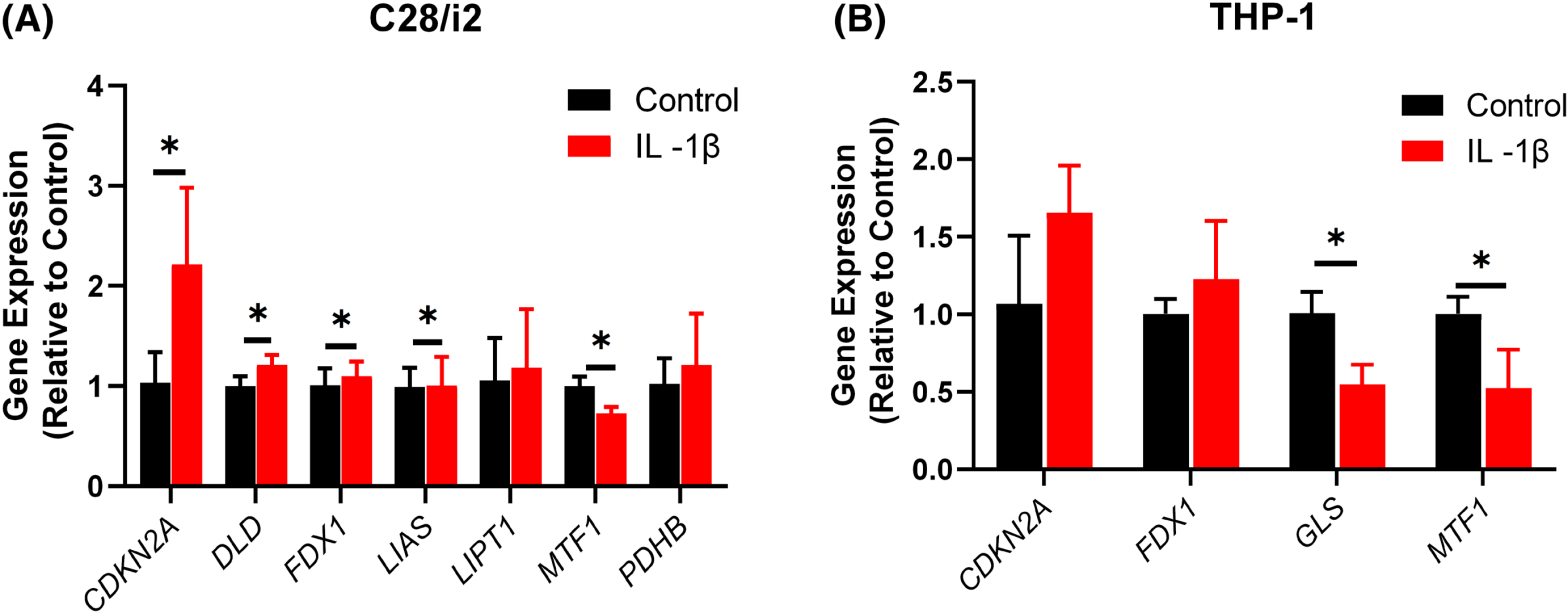
Investigation of CRGs expression in C28/i2 chondrocyte and THP‐1 monocyte. (A) The expression levels of CRGs in C28/i2 chondrocyte by RT‐PCR. (B) The expression levels of CRGs in THP‐1 monocyte. All data were presented as mean ± SD, *p < 0.05, **p < 0.01, ***p < 0.001.

We exposed C28/i2 chondrocytes to a stimulation of 10 ng/mL IL‐1β and analysed the expression levels of CRGs. The result showed that these expression levels of *CDKN2A*, *DLD*, *FDX1*, *LIPT1* and *PDHB* in IL‐1β stimulation group were higher more than the control group, expect *MTF1* (Figure 8A). We further cultured monocytes using conditioned media (with or without IL‐1β), and then extracted total RNA to verify the expression levels of *CDKN2A*, *FDX1*, *GLS* and *MTF1*. The result showed that the expression level of *CDKN2A* and *FDX1* were significantly increased in monocytes under IL‐1β stimulation, while the expression level of *GLS* and *MTF1* were significantly decreased (Figure 8B). These experimental results are consistent with the data analysis.

### 3.7 Validation of cuproptosis in chondrocyte under IL‐1β stimulation or excess copper

**FIGURE 9:**
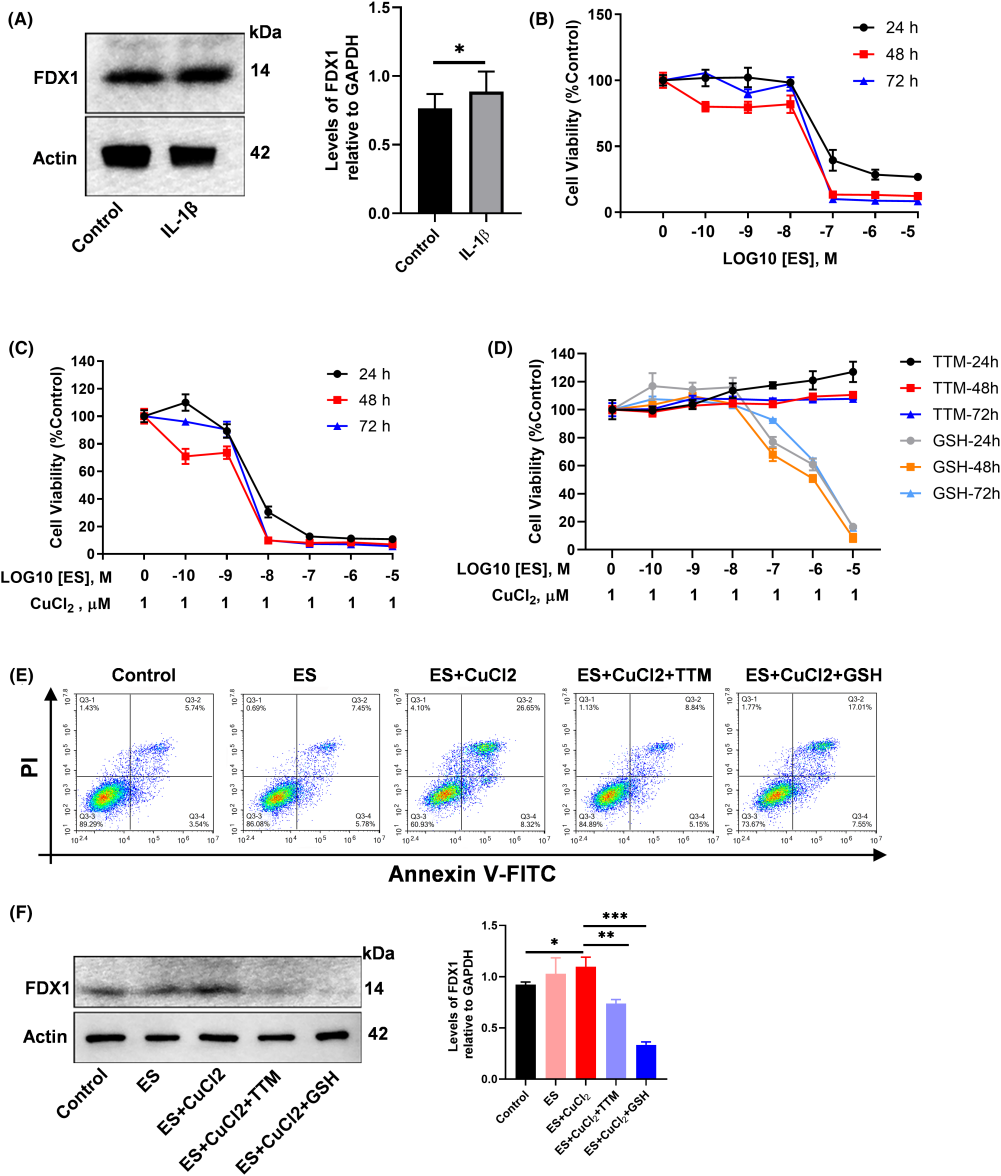
Validation of cuproptosis in chondrocyte under IL‐1β stimulation or excess copper. (A) The expression level and statistical analysis of FDX1 in C28/i2 chondrocyte with or without 10 ng/mL IL‐1β was assessed. (B) The impact of and cuproptosis activitor (Elesclomol) on chondrocyte viability was assessed using the CCK‐8 assay. (C) The impact of and Elesclomol added 1 μM CuCl_2_ on chondrocyte viability was assessed using the CCK‐8 assay. (D) The impact of and Elesclomol added 1 μM CuCl_2_ with 10 μM TTM or 1 mM GSH on chondrocyte viability was assessed using the CCK‐8 assay. (E) The apoptosis of C28/i2 induced by 10^−8^ M ES, 10^−8^ M ES + 1 μM CuCl_2_, 10^−8^ M ES + 1 μM CuCl_2_ + 10 μM TTM, 10^−8^ M ES + 1 μM CuCl_2_ + 1 mM GSH for 48 h was assessed by Flow cytometry. (F) The expression level and statistical analysis of FDX1 in C28/i2 chondrocyte induced by 10^−8^ M ES, 10^−8^ M ES + 1 μM CuCl_2_, 10^−8^ M ES + 1 μM CuCl_2_ + 10 μM TTM, 10^−8^ M ES + 1 μM CuCl_2_ + 1 mM GSH for 48 h. All data were presented as mean ± SD, *p < 0.05, **p < 0.01, ***p < 0.001.

To investigate the role of cuproptosis in the pathogenesis of OA, we conducted experiments on chondrocytes by 10 ng/mL IL‐1β stimulation to monitor changes in FDX1 expression. The experimental findings indicate that IL‐1β significantly upregulated the expression level of FDX1 in chondrocytes (Figure 9A, Figure S2A). Furthermore, we treated the chondrocytes with a cuproptosis promoter (Elesclomol, ES) under both Cu^2+^ supplemented and unsupplemented conditions to assess its impact on cellular viability and FDX1 expression. These results revealed that the ES at a concentration of 10^−8^ M under both Cu^2+^ supplemented conditions significantly decrease cell viability compared with Cu^2+^ unsupplemented conditions (Figure 9B,C, Figure S2B,C). To further verify the impact of cuproptosis on chondrocyte viability, we utilized a combination of the copper ion chelator TTM (10 μM, Figure S1) and the reducing agent GSH (1 mM), treating cells under conditions of 10^−8^ M ES and 1 μM CuCl_2_. The experimental results demonstrated that 10 μM TTM could completely prevent the reduction in cell viability induced by ES + CuCl_2_, whereas 1 mM GSH showed a gradual decline in its copper ion chelation efficacy when the ES concentration exceeded 10^−7^ M, resulting in decreased cell viability (Figure 9D, Figure S2D). Flow cytometry analysis revealed that the ES + CuCl_2_ combination significantly induced apoptosis, while TTM inhibited apoptosis under these conditions. In contrast, GSH failed to inhibit apoptosis (Figure 9E, Figure S2E). Detection of FDX1 expression levels indicated that the expression in the ES + CuCl_2_ group was significantly higher than in the control group, whereas the FDX1 expression levels in the ES + CuCl_2_ + TTM and ES + CuCl_2_ + GSH groups were markedly lower than in the ES + CuCl_2_ group (Figure 9F). These findings suggest that IL‐1β stimulation of chondrocytes may induce cuproptosis, and that cuproptosis activator can significantly promote chondrocyte cuproptosis.

## 4 DISCUSSION

OA is prevalently observed in the middle‐aged and elderly population. Epidemiological data suggest that in individuals aged 60 and above, approximately 10% of males and 18% of females experience symptoms caused by OA, with females presenting a higher incidence rate of structural OA compared to males.^3^, ^44^ OA is a complex disease involving the interplay of multiple genes and pathways. The pathogenesis encompasses a variety of factors, including growth factors, inflammatory cytokines and the signalling molecules involved in these processes.^45^ In this study, we initially employed bioinformatics approaches to identify seven differentially expressed CRGs that are pivotal for chondrocyte apoptosis in the progression of OA, specifically including *CDKN2A*, *DLD*, *FDX1*, *LIAS*, *MTF1*, *LIPT1* and *PDHB*. During the OA process, cartilage tissue is infiltrated by a multitude of immune cell types. Further analysis revealed a close correlation between the extent of immune cell infiltration and the expression levels of CRGs. Notably, the expression of the *LIAS* gene was associated with the infiltration of almost all types of immune cells. Moreover, we confirmed that the expression of CRGs in chondrocytes and monocytes was significantly altered under inflammatory conditions induced by IL‐1β, and that cuproptosis inducers triggered apoptosis in chondrocytes and elevated FDX1 expression levels. These findings pave the way for future exploration of the pathogenesis and therapeutic targets for OA.

Copper is an essential trace element in human physiology with profound effects on various biological processes, including erythropoiesis, mitochondrial function, neurotransmitter metabolism, redox homeostasis and extracellular matrix equilibrium.^13^ In maintaining joint health, the metabolic balance of copper plays a critical role. A deficiency in copper can lead to impaired activity of various enzymes that depend on copper ions as cofactors, subsequently affecting collagen synthesis and rendering cartilage more susceptible to fragility, ultimately jeopardizing the structural integrity of cartilage.^16^, ^46^ Excessive copper can induce alterations in the microfilaments, microtubules and fibrous structures of cartilage cells, which can interfere with the normal formation of the cartilage cell skeleton.^47^ Furthermore, a clear correlation exists between excessive accumulation of copper and cartilage damage, although the specific mechanisms remain unclear. Cuproptosis has been discovered in the development of various diseases, including OA.^12^, ^14^ Excess copper accumulation in cells can cause lipid modification damage in mitochondria, hinder the function of key enzymes in the tricarboxylic acid (TCA) cycle, and disrupt the formation of Fe‐S clusters, leading to cell death, a process referred to as cuproptosis.^20^ Remarkably, research on OA has confirmed that the copper content in biological samples of affected individuals is significantly increased.^11^, ^18^ Additionally, Mendelian randomization analysis has also confirmed that elevated serum copper levels are associated with an increased susceptibility to OA.^48^ These findings suggest that cuproptosis plays a role in the development of OA. In this study, we analysed the expression of 10 CRGs and found significant differences in the expression of five cuproptosis‐activating genes (*DLD*, *FDX1*, *LIAS*, *PDHB* and *LIPT1*) and two cuproptosis‐inhibitory genes (*CDKN2A* and *MTF1*) between OA patients and a healthy population. Based on these findings, we constructed a model to predict clinical OA, discovering that the expression of *FDX1* and *LIPT1* (cuproptosis‐activating genes), as well as *CDKN2A*, *GLS* and *MTF1* (cuproptosis‐inhibitory genes), was significantly associated with the occurrence of OA, consistent with previous research.^20^ Moreover, in IL‐1β‐stimulated chondrocytes, we validated the expression levels of these CRGs via qRT‐PCR, with results aligning with predictions. Particularly, FDX1, as a key regulatory protein in cuproptosis,^30^ exhibited a significant increase in expression in chondrocytes under the influence of IL‐1β, indicating that IL‐1β could promote cuproptosis. Additionally, when chondrocytes were exposed to cuproptosis activitor, the cells underwent apoptosis and the expression of FDX1 concurrently increased, further illustrating the role of cuproptosis in this process.

In the pathogenesis of OA, immune cell infiltration plays a crucial role. This study employs unsupervised clustering analysis, utilizing multiple datasets (GSE12021, GSE55235 and GSE55457), to explore the expression patterns of CRGs in OA patients, providing a scientific basis for developing immunomodulatory treatment strategies for OA. The results indicate that there are significant differences in the distribution of different clusters across the datasets, suggesting that the origin of the datasets may influence the clustering outcomes. However, our primary finding—the significant association between clusters and 10 CRGs—was validated across all datasets. This indicates that despite potential confounding factors due to dataset origins, the biological importance and robustness of the relationship between clusters and CRGs are maintained. Furthermore, except for the *DLD*, *DLAT* and *PDHA1* genes, other CRGs were significantly correlated with immune cell infiltration, with the *LIPT1* gene showing a universal correlation with various types of immune cell infiltration. The expression of *CDKN2A*, *FDX1*, *GLS* and *MTF1* genes was validated in conditionally cultured monocytes, aligning with bioinformatics analyses. This further underscores the significant statistical differences in immune cell abundance among clusters, despite the differences between datasets. This also corroborates the biological significance of the relationship between clusters and CRGs. This finding reinforces the reliability of our predictive model for OA in clinical diagnostics. However, to gain a more comprehensive understanding of these results, future work should focus on two aspects: firstly, when integrating multi‐center data, evaluating the potential impact of different datasets on the analysis results and employing multivariable adjustments and stratified analyses in study design to mitigate these confounding factors. Secondly, further experimental validation and clinical sample analysis are needed to support our findings.

## 5 CONCLUSION

Our investigation encompassed an examination of the gene expressions associated with cuproptosis in OA, exploration the correlation between these genes and immune infiltration, and validation of key protein expressions involved in cuproptosis during chondrocyte apoptosis. Our findings unveiled a positive association between the expression of *FDX1* and *LIPT1* genes and the occurrence of OA, while *CDKN2A*, *GLS* and *MTF1* displayed a negative correlation. Furthermore, we observed a relationship between the abundance of specific immune cells in the synovium and the expression of CRGs, particularly *LIPT1*. Notably, the most significant discovery of our study manifested through a noteworthy elevation in the expression level of FDX1, a key cuproptosis protein, during chondrocyte apoptosis, as well as the promotion of chondrocyte apoptosis by cuproptosis inducers. The above demonstrates that our predictive model holds significant reference value in clinical diagnosis. However, we acknowledge certain limitations in our study. Primarily, the data utilized originated from databases, which may introduce potential biases and limitations in terms of sample size and quality. Moreover, our validation experiments were solely conducted at the cellular level, lacking further in vivo and clinical verifications. Therefore, the generalizability and clinical relevance of our findings should be interpreted with caution. Additionally, our focus was solely on confirming the occurrence of cuproptosis in chondrocyte apoptosis, without addressing its immunological implications. Nonetheless, our present results furnish preliminary evidence that targeting copper‐mediated cell demise may offer promising avenues for advancing our understanding of OA pathogenesis and the development of therapeutic interventions.

## AUTHOR CONTRIBUTIONS

**Jingmin Che:** Conceptualization (equal); data curation (lead); formal analysis (equal); methodology (equal); software (equal); visualization (equal); writing – original draft (lead). **Xiaoli Yang:** Data curation (equal); methodology (equal); software (equal); visualization (equal). **Xiangrong Zhao:** Data curation (equal); methodology (equal); validation (equal). **Yan Li:** Data curation (equal); methodology (equal). **Zhankui Jin:** Conceptualization (equal); funding acquisition (lead); project administration (equal); writing – review and editing (equal). **Cuixiang Xu:** Conceptualization (equal); project administration (equal); resources (equal); writing – review and editing (equal).

## FUNDING INFORMATION

This work was supported by the Natural Science Basic Research Program of Shaanxi Province, grant number 2023‐JC‐YB‐790, and the Technology Talent Support Program of Shaanxi Provincial People's Hospital, grant number 2022BJ‐04.

## CONFLICT OF INTEREST STATEMENT

The authors declare no conflict of interest.

## CONSENT FOR PUBLICATION

All authors consent to the publication of this study.

## ETHICS STATEMENT

Not applicable.

## Supporting information


Data S1.

## Data Availability

All data associated with this study are present in the paper or the Supplementary Materials. Requests for data should be addressed to the corresponding authors.
