# Supplementary material for: Risk factor prediction and immune correlation analysis of cuproptosis‐related gene in osteoarthritis
Source: J Cell Mol Med. 2024 Aug 1;28(15):e18574. doi: 10.1111/jcmm.18574 (PMC11292577; doi:10.1111/jcmm.18574)
Supplement: Supplementary file 1 — Data S1. [file JCMM-28-e18574-s001.docx]

Supplement figure 1


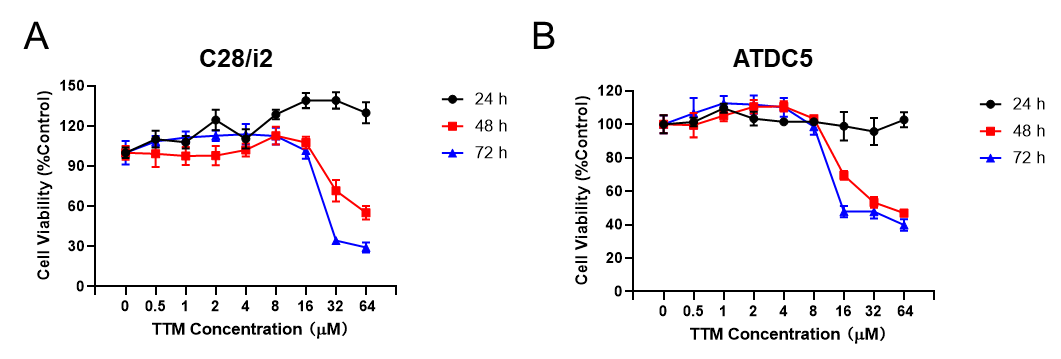


**(A)** The impact of copper ion chelator—TTM on C28/i2 chondrocyte viability was assessed using the CCK-8 assay. **(B)** The impact of copper ion chelator—TTM onATDC5 chondrocyte viability was assessed using the CCK-8 assay.

Supplement figure 2


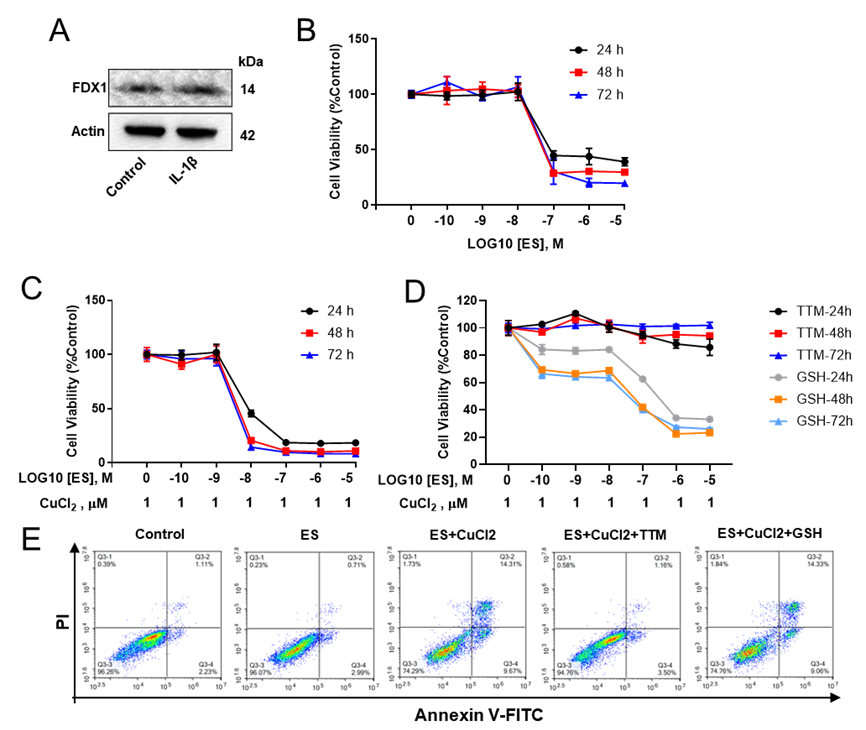


**Validation of cuproptosis in ATDC 5 chondrocyte under IL-1β stimulation or excess copper.** **(A)** The expression level and statistical analysis of FDX1 in ATDC5 chondrocyte with or without 10 ng/mL IL-1β was assessed. (B) The impact of and cuproptosis activitor (Elesclomol) on chondrocyte viability was assessed using the CCK-8 assay. (C) The impact of and Elesclomol added 1μM CuCl_2_ on chondrocyte viability was assessed using the CCK-8 assay. (D) The impact of and Elesclomol added 1μM CuCl_2_ with 10 μM TTM or 1 mM GSH on chondrocyte viability was assessed using the CCK-8 assay. (E) The apoptosis of ATDC5 chondrocyte induced by 10^-8^M ES, 10^-8^M ES+1μM CuCl_2_, 10^-8^M ES+1μM CuCl_2_+10 μM TTM, 10^-8^M ES+1μM CuCl_2_+1 mM GSH for 48 h was assessed by Flow cytometry.
